# Olfactory coding from the periphery to higher brain centers in the *Drosophila* brain

**DOI:** 10.1186/s12915-017-0389-z

**Published:** 2017-06-30

**Authors:** Yoichi Seki, Hany K. M. Dweck, Jürgen Rybak, Dieter Wicher, Silke Sachse, Bill S. Hansson

**Affiliations:** 1 Present address: Laboratory of Molecular Neuroscience and Neurology, School of Life Sciences, Tokyo University of Pharmacy and Life Sciences, 1432-1 Horinouchi, Hachioji, Tokyo, 192-0392 Japan; 2 Present address: Department of Molecular, Cellular, and Developmental Biology, Yale University, New Haven, CT 06520 USA; 3 Department of Evolutionary Neuroethology, Max Planck Institute for Chemical Ecology, Hans-Knöll-Strasse 8, 07745 Jena, Germany

**Keywords:** *Drosophila melanogaster*, Insect olfaction, Antennal lobe, Mushroom body, Lateral horn, Whole cell patch-clamp

## Abstract

**Background:**

Odor information is processed through multiple receptor-glomerular channels in the first order olfactory center, the antennal lobe (AL), then reformatted into higher brain centers and eventually perceived by the fly. To reveal the logic of olfaction, it is fundamental to map odor representations from the glomerular channels into higher brain centers.

**Results:**

We characterize odor response profiles of AL projection neurons (PNs) originating from 31 glomeruli using whole cell patch-clamp recordings in *Drosophila melanogaster*. We reveal that odor representation from olfactory sensory neurons to PNs is generally conserved, while transformation of odor tuning curves is glomerulus-dependent. Reconstructions of PNs reveal that attractive and aversive odors are represented in different clusters of glomeruli in the AL. These separate representations are preserved into higher brain centers, where attractive and aversive odors are segregated into two regions in the lateral horn and partly separated in the mushroom body calyx.

**Conclusions:**

Our study reveals spatial representation of odor valence coding from the AL to higher brain centers. These results provide a global picture of the olfactory circuit design underlying innate odor-guided behavior.

**Electronic supplementary material:**

The online version of this article (doi:10.1186/s12915-017-0389-z) contains supplementary material, which is available to authorized users.

## Background

A common feature of the olfactory system in insects, mammals, and most other higher animals possessing a sense of smell is an organization where the first order olfactory center (the antennal lobe (AL) and the olfactory bulb) comprise spherical neuropils called glomeruli, each of which receives convergent input from olfactory sensory neurons (OSNs) expressing a given odorant receptor (OR) [1, 2]. Most odors are detected by multiple olfactory receptor classes with various binding affinities. This enables animals to discriminate many more odors than the number of receptor classes expressed. However, although one of the fundamental questions in olfaction is to understand the central processing underlying such combinatorial coding, the answer still remains elusive.

**Fig. 1:**
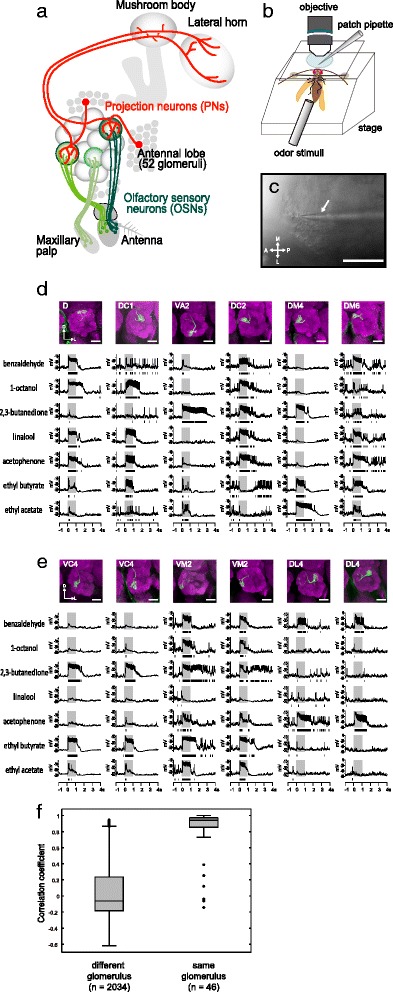
In vivo whole cell patch-clamp recordings from antennal lobe (*AL*) projection neurons (*PNs*). **a** Schematic diagram of the *Drosophila* olfactory pathway from the periphery to higher brain centers. **b** Schematic diagram of a fly preparation for in vivo whole cell patch-clamp recording (for details see Methods). **c** Recordings were obtained from a cell body of a PN. The glass pipette is indicated by the *arrow*. Infrared differential interference contrast (*DIC*) view. Scale bar = 50 μm. **d** Example traces of odor responses of six different class PNs innervating different glomeruli. The *gray bars* indicate a 1-s odor stimulus period. Raster plots of the spikes are shown under the traces. Morphology of the PNs (*top row*) showing the dendritic innervation to a glomerulus in the AL in a projection of a confocal stack (PN dendrites: *green*, nc82: *magenta*). Scale bar = 20 μm. **e** Example traces of odor responses of three pairs of PN classes innervating the same glomerulus. The *gray bars* indicate a 1-s odor stimulus period. Raster plots of the spikes are shown under the traces. Morphology of the PNs (*top row*) showing the dendritic innervation to a glomerulus in the AL in a projection of a confocal stack (PN dendrites: *green*, nc82: *magenta*). Scale bar = 20 μm. **f** Pearson's correlation coefficient of odor responses between PN pairs innervating the different glomeruli or the same glomerulus

The olfactory system of *Drosophila melanogaster* has become an excellent model system to study and to understand the neuronal processing of olfactory information. The neuromorphological organization of the *Drosophila* AL circuitry has been extensively studied [3, 4]. The axons of OSNs expressing the same olfactory receptor converge onto one of the 52 AL glomeruli [5–8]. There they form synapses with two types of projection neurons (PNs): one type consists of cholinergic (i.e., excitatory) PNs that mainly receive input in a single glomerulus and relay the information to the mushroom body (MB) calyx and the lateral horn (LH) (Fig. 1a), while the other PN type represents GABAergic (i.e., inhibitory) PNs that mainly innervate multiple glomeruli and send their axons directly to the LH [9–15]. Several studies analyzing the anatomical map of axonal projections of PNs in the MB calyx and the LH have shown less deterministic wiring patterns in the MB calyx and highly stereotyped projection patterns in the LH [11, 13, 16, 17]. This supports the view that the MB is involved in plastic processes such as learning and memory, while the LH is directly linked to innate behaviors [13, 18–22].

The *Drosophila* and the vertebrate olfactory system share a similar organization from the first order processing areas up to higher brain centers [23, 24]. In mice, axonal projections of mitral and tufted cells (equivalent to PNs) from individual glomeruli spread diffusely to the piriform cortex without apparent spatial preference, while they show relatively stereotyped innervation to the cortical amygdala. Since the piriform cortex is associated with olfactory learning, and the cortical amygdala plays an important role in innate behavior, this dichotomous organization resembles the MB and LH structures in insects [23, 24]. Comparing such similarities and differences between vertebrates and insects helps in shedding light on general principles of the olfactory system.

How does a fly interpret odor information? Previous studies have revealed a relationship between odor representations in the AL and innate behaviors [25]. Several ecologically relevant odors use a dedicated pathway leading to an innate behavior, such as the male-produced pheromone 11-*cis*-vaccenyl acetate (cVA) [26, 27], the mating enhancing pheromone methyl laurate [28], CO_2_ [29], acids [30], ammonia and amines [22], parasitoid odors [31], and the microbial odorant geosmin [32]. In contrast, other odors that also induce innate attraction or aversion are processed via a combinatorial code comprising multiple glomeruli. For instance, a highly attractant mixture induces a specific activation pattern among a combination of glomeruli [33]. Knaden et al. screened 110 monomolecular odorants for their innate odor valence, thereby revealing a valence-related spatial representation often comprising combinatorial glomerular activation patterns [34]. Although another study demonstrated that individual glomeruli, rather than the entire pattern of active glomeruli, mediate innate olfactory attraction and aversion [35], a recent study demonstrated that flies’ innate odor responses can be predicted by a model summing normalized glomerular responses, in which each glomerulus contributes a specific, small amount to odor preference [36]. How does such odor information of different innate valence converge and interact at higher brain centers? Without a precise neural map and a functional map to place on top of it, it is difficult to assess how glomerular activation patterns would be integrated into higher brain centers.

In this study, we used in vivo whole cell patch-clamp recordings in *Drosophila melanogaster* and mapped odor response profiles of 71 PNs emerging from 31 glomeruli (covering 60% of ~50 glomeruli in total) to 17 different odors including innately attractive and aversive odors. We addressed the following questions: How is odor information transformed from OSNs to PNs? How is odor information mapped at the level of AL glomeruli and transformed into higher brain centers? And, finally, how is this map related to the fly’s perception and innate behavior?

## Results

### Many odors are encoded by PNs in a combinatorial manner

We characterized odor response profiles of 71 PNs using in vivo whole cell patch-clamp recordings and subsequently stained them to identify the innervated glomerulus for each individual neuron (Fig. 1b–d). A total of 67 out of the recorded 71 PNs represent uniglomerular PNs that extended their primary dendrites into a single glomerulus and sent their axons to the calyx of the MB and the LH through the medial antennal lobe tract (mALT) also known as the inner antenno-cerebral tract (IACT) (Fig. 1a). The remaining four PNs innervated either two glomeruli or the region posterior to the AL (data not shown). In this study, we focused our analyses on the 67 uniglomerular PNs innervating one out of 31 glomeruli and therefore covering ~60% of 52 olfactory glomeruli present in the *Drosophila* AL. We tested 17 behaviorally relevant monomolecular odors that flies encounter under natural conditions (Additional file 1: Table S1). These odors induce innate attractive or aversive behaviors, which was earlier demonstrated using diverse behavioral assays, such as the trap assay [34], the T-maze assay [13, 32, 37], or the FlyWalk [38, 39]. We defined the 17 odors as attractive or aversive if flies showed attractive or aversive odor-guided behavior toward these odors in at least one of the behavioral assays (see Additional file 1: Table S1).

PNs showed a spike firing increase when activated by an odor (Fig. 1d). To evaluate odor-induced neural activities, we quantified spike numbers during the 1-s odor stimulus as an indicator of odor response intensity. We observed some slight variations in odor-induced temporal firing patterns, such as lengths of firing phases (Fig. 1d, e), but confined our analysis to the odor response intensity emphasizing rate coding on the system. Temporal patterns might convey critical information on odor stimulus features as demonstrated in other olfactory systems [40], but we address this issue later (see the section on Temporal dynamics of odor representation).

There are in total ~150 uniglomerular PNs and 52 glomeruli; the number of PNs innervating the same glomerulus is on average three but is variable between glomeruli in that the glomeruli innervated by narrowly tuned OSNs seem to possess a larger number of PNs, while the glomeruli innervated by broadly tuned OSNs possess a smaller number of PNs [4, 41, 42]. A previous study reported that these sister PNs have highly correlated patterns of activity in odor responses as well as spontaneous synaptic inputs [43]. Therefore, it can be assumed that sister PNs should be identical in terms of odor responses, thereby resulting in ~50 PN classes. In the present study, we investigated one PN per fly; therefore, it was necessary to confirm that odor responses of each PN class would be reproducible under our recording conditions, even when recorded in different individuals. Indeed we found that odor response profiles of the same PN class (i.e., innervating the same glomerulus) were highly similar (Fig. 1e). The degree of variation in the odor responses of the same PN class is shown in Additional file 2: Figure S1, illustrating the caveat that although the response patterns were similar, there was inter-neuron variability in the magnitude of odor responses among the same class of PNs. Furthermore, we calculated the Pearson’s correlation coefficient based on odor response intensities to 17 odors between the pairs of all PNs and corroborated that the responses in pairs of PNs innervating the same glomerulus were highly correlated (median *r* = 0.9402, *n* = 46), while the responses in pairs of PNs innervating different glomeruli were not (median *r* = –0.0634, *n* = 2034, *p* < 10^–24^, Wilcoxon rank sum test; Fig. 1f). From this we concluded that characteristics of PNs investigated in different flies could be directly compared.

**Fig. 2:**
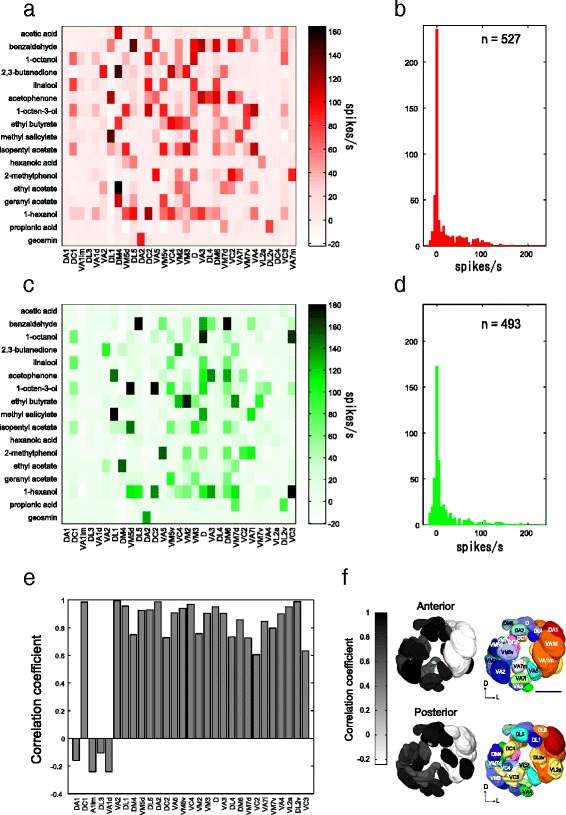
Comparison of odor responses between PNs and OSNs. **a** The summary of odor response intensity of 31 PN classes to 17 odors. Spike frequencies during a 1-s odor stimulation period are color coded. **b** Histograms of PN response intensity. The histogram is accumulated across all 527 PN-odor pair response magnitudes (31 PN classes × 17 odors). **c** The summary of odor response intensity of 29 OSN classes to 17 odors recorded by single sensillum recordings. Spike frequencies during a 1-s odor stimulation period are color coded. **d** Histograms of OSN response intensity. The histogram is accumulated across all 493 OSN-odor pair response magnitudes (29 OSN classes × 17 odors). **e** Pearson's correlation coefficient between PN and OSN odor response intensities compared for each of 29 glomeruli. **f** The same values of Pearson's correlation coefficient as in **e** are mapped on the 29 glomeruli in a template AL

We characterized the odor response profiles of 31 PN classes from 67 PN recordings using in vivo whole cell patch-clamp recordings (Fig. 2a, Additional file 3: Table S2). The data obtained from the different PN classes revealed that many odors activated more than one PN class and most PNs showed an increased spiking rate to more than one odor, except for a few PN classes (Fig. 2a). Hence, many odors are encoded by PNs in a combinatorial manner at an odor concentration of 10^–3^, thereby confirming previous studies (e.g., [44]). However, strong responses were sparse: by comparing all odor-PN class pairs, only 16.7% (*n* = 88/527) exceeded the response of ≥50 spikes during the 1-s odor stimulus and 22.8% (*n* = 120/527) exceeded the response of 30 spikes/s (Fig. 2b). In addition, a few PN classes did not show any clear responses (responses were ≤10 spikes/s) to any odors in our odor set (e.g., PNs innervating glomeruli DA1 and DL3).

### A global picture of odor transformation between OSNs and PNs

How is the odor information transformed from OSNs to PNs? A previous study comparing neurons innervating seven glomeruli revealed that odor tuning curves tend to be broader in PNs than in the corresponding OSNs that innervate the same glomerulus [45]. By contrast, narrowly tuned odor response profiles are conserved between OSNs and PNs in the case of dedicated channels mediating odors of specific ecological significance [28, 32, 46]. As these investigations point in different directions, we asked if there is a general rule for odor transformation using our set of 17 odors recorded in PNs innervating more than half of the AL glomeruli.

First, in order to characterize the odor response profiles of OSNs corresponding to our PN recordings, we performed single sensillum recordings (SSRs). In *D. melanogaster*, 17 types of sensilla have been described, including trichoid, basiconic, and coeloconic types; each houses one to four OSN classes [5, 47]. Via a system-wide SSR screen from all OSN classes [32, 48], we were able to characterize odor response profiles of 29 OSN classes corresponding to 29 of the 31 glomeruli that were covered by our PN recordings (*n* = 3 for each OSN class, Fig. 2c, Additional file 4: Table S3). Odor response profiles of two OSN classes could not be characterized; one OSN class projecting to DC4 is housed in grooved sensilla that project to the interior of the sacculus that are not accessible by SSR [30], and the other OSN class projecting to VA7m has not been assigned to a specific functional class [5]. Our SSRs show that many odors activate more than one OSN class and many OSN classes respond to more than one odor (Fig. 2c). There are thus similar combinatorial patterns as observed for the PN recordings. Only 13.8% of the odor-OSN pairs investigated (*n* = 68/493) showed ≥50 spikes during the 1-s odor stimulus and 18.6% (*n* = 90/473) exceeded ≥30 spikes to the set of odors tested in the present study (Fig. 2d).

Next, we reconstructed spatial odor response maps of OSNs and PNs in the AL and compared these maps with each other (Additional file 5: Figure S2). We found a remarkable similarity between these two maps, suggesting that odor response patterns are fairly conserved between the two processing levels. Then, to evaluate how odor tuning is transformed from OSNs to PNs in the same glomeruli, we calculated the Pearson’s correlation coefficient based on odor response intensities to the 17 odors between the corresponding OSN and PN classes for the 29 glomeruli, where both OSN and PN odor response profiles were available. The correlation was moderately high (*r* = 0.72 ± 0.38, *n* = 29, *p* < 0.01 for the 25 glomeruli except for DA1, VA1lm, DL3, and VA1d, Pearson’s correlation; Fig. 2e, f). When the OSN and PN glomerular combinations were randomized, the high correlation was dramatically decreased (*r* = 0.14 ± 0.038, *n* = 29, 100 runs). Notably, for the four glomeruli DA1, VA1lm, DL3, and VA1d, a low correlation was found (*r* = 0.19 ± 0.07, *n* = 4, *p* > 0.1 for all four glomeruli, Pearson’s correlation). These glomeruli are innervated by OSNs present in trichoid sensilla, and the specific ligands for the OSNs and PNs targeting these were not included in our odor set [28, 49]. Except for these low response glomeruli, we found a similar range of correlation (*r* ^2^ = 0.37–0.99) in the 25 glomeruli as previously reported (*r* ^2^ = 0.26–0.81 in the 7 glomeruli [45]), supporting the idea that odor response profiles are generally conserved between OSNs and PNs, but likely modulated by lateral interactions between glomeruli [50, 51].

**Fig. 3:**
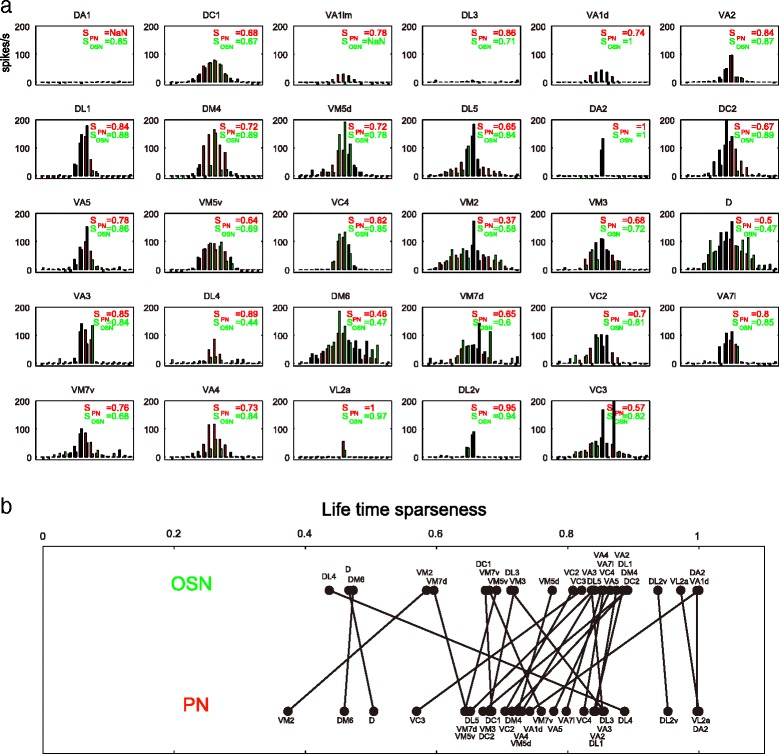
Tuning curve transformation varies depending on the glomeruli. **a** Tuning curves for PNs (*red*) and OSNs (*green*). The order of 17 odor stimuli along the *x* axis is arranged according to PN responses so that the strongest responses are placed on the center and the weakest responses are placed near the edges. The value of lifetime sparseness is indicated in each graph. **b** Lifetime sparseness of corresponding OSNs and PNs is calculated in the 27 glomeruli (among 29 glomeruli except for DA1 and VA1lm glomeruli)

Finally we calculated the lifetime sparseness as a quantitative measure for the odor tuning curves observed and compared these values between OSNs and PNs innervating the same glomerulus [44, 45] (Fig. 3a). In some glomeruli, odor tuning curves indeed became broader in PNs as compared to the cognate OSNs, whereas in other glomeruli only small changes of odor tuning curves were observed or tuning curves became even narrower (Fig. 3b). These results, contrary to earlier hypotheses, suggest that transformations of odor tuning curves are complex and vary in a glomerulus-dependent manner.

Our results thus provide a global perspective of the transformation in the AL by covering more than half of the glomeruli and their innervating input and output elements. Further, we reveal a non-uniform trend of odor tuning transformation between OSNs and PNs. As in most olfactory studies, a general caveat is that our odor set included only 17 odors, which will inevitably cause some bias. We do, however, know that these odors are behaviorally significant to the fly.

### A functional odor representation map in the AL

**Fig. 4:**
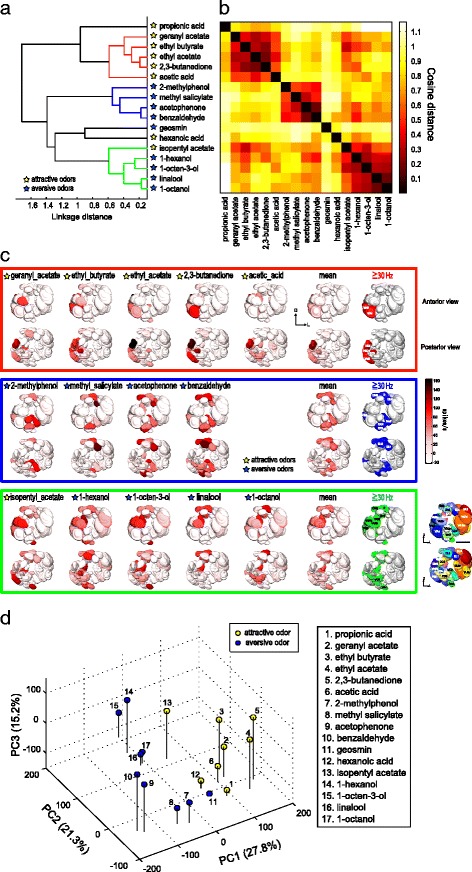
Odors are separately represented by different combinations of glomeruli. **a** Hierarchical cluster analysis for 17 odors based on the cosine distances between odor response intensities of 31 PN classes. The cut-off threshold is set at 0.6 linkage distance, detecting three groups of separately clustered odors colored in *red*, *blue*, and *green*. **b** A complete distance matrix measured with cosine distances for 17 odors based on odor response intensities of the 31 PN classes. Each axis of the matrix is ordered as in **a**. **c** Spatial response patterns for odors clustered in the three clusters in **a** using odor response intensity of 31 PN classes on a template AL. Mean activation patterns using the average of the odor representation within the same cluster and glomeruli extracted by setting a threshold of 30 spikes/s are shown for three clusters in the right column. Anterior view of the AL (*top*) and posterior view of the AL (*bottom*). **d** Principal component analyses for 17 odors based on the odor response intensities of the 31 PN classes. The percentages of variance accounted by each PC component are shown on each axis

Our results confirmed that most odors are encoded by a combinatorial pattern involving multiple glomeruli. Each odor can thus be represented in a multidimensional odor space with each glomerular channel representing a single dimension. We proceeded to investigate how odors can be classified by the multiple glomerular channels investigated. In our dataset, each odor can be denoted by an odor response vector consisting of the odor response intensities of the 31 PN classes to the odor. A distance of two odors can be evaluated by calculating a distance between the two odor response vectors. In a previous study, a close relationship between odor discrimination and ensemble PN population activity was demonstrated, where the distances between two odors were evaluated by using cosine or Euclidean distances of PN population activities, and where these distances could account for the degree of odor discrimination between odors [21]. These two metrics could thus be suitable for evaluating odor representation of the ensemble PN population activities. The difference between the two distance metrics is that the cosine distance is insensitive to amplitude, thereby emphasizing the patterns of PN activities, while the Euclidean distance is sensitive to amplitude, thus emphasizing absolute response intensities. First, we performed a hierarchical cluster analysis for our 17 odors based on response intensities of the 31 PN classes using cosine distances (Fig. 4a, b). We found that three groups of odors were classified (Fig. 4a, b): The first cluster included five odors including three esters, a ketone, and an acid, all of which are attractive to the fly (Fig. 4a; colored in red). The second cluster included four aversive odors that are all aromatic compounds (Fig. 4a; colored in blue). The third cluster included four aversive odors (a terpene and three alcohols) and one attractive odor (an ester) (Fig. 4a; colored in green). The reconstructed activity patterns induced by the odors in the first cluster showed that glomeruli activated by these odors were biased to the medial side of the AL, while those activated by odors in the second cluster comprised several glomeruli located in the dorsal and ventral regions of the AL, and those activated by odors in the third cluster comprised dorsal and anterior-central glomeruli (Fig. 4c). Notably, the odor-specific patterns often comprised neighboring glomeruli. First, we confirmed this by examining the cluster-specific patterns by averaging odor response intensities among the odors within the same cluster and extracting the glomeruli that mainly contributed to forming the cluster-specific patterns (by setting the threshold of the mean spike rate at 30 spikes/s) (Fig. 4c). These patterns were not completely distinct, as there was a small overlap between the different patterns such that the VM2 glomerulus was included in all three patterns, while glomeruli D and DM6 were included in both aversive odor patterns (Fig. 4c). Second, we calculated the correlation between glomerular anatomical distances and the PN odor response similarity using cosine distances for each pair of the 31 glomeruli (Additional file 6: Figure S3). We found a moderate correlation (*r* = 0.232; *p* = 4.22 × 10^–7^). When the labels between the pairs of glomeruli and their anatomical distances were randomized, this correlation disappeared (*r* = 0.0038 ± 0.3532, 100 runs). This result suggests that there is an anatomical arrangement, where neighboring glomeruli tend to have similar odor response properties, thus corroborating the clustering arrangement of glomeruli.

We also performed a hierarchical cluster analysis for our 17 odors based on response intensities of the 31 PN classes using Euclidean distances (Additional file 7: Figure S4). Similar divisions were obtained as when using the cosine distance, thereby confirming that the odors can be classified into three groups. However, since the Euclidean distances depend on absolute intensities of odor response, one cluster appeared to be formed partly based on the low response intensities elicited by these odors (Additional file 7: Figure S4a, colored in red).

We next applied a principal component analysis (PCA) to visualize the 31-dimensional odor space in three dimensions (Fig. 4d). Consistent with the hierarchical cluster analysis, we found that attractive and aversive odors clustered separately. The PC1, accounting for 27.8% data variance, roughly separated attractive and aversive odors, as demonstrated by a previous study (Knaden et al. 2012) [34], and the PC2 (21.3%) further separated the two aversive odor groups. In summary, these results indicate that attractive and aversive odors are significantly separately represented by different combinations of glomeruli (*p* < 0.001, one-way analysis of similarities, ANOSIM).

### Implication of the AL functional map

By measuring the distances among odors in PN neural space, we found three odor clusters that were represented by separate, but partly overlapping, groups of neighboring glomeruli (Fig. 4a–c). Since we have shown that the odors within the same cluster induced similar glomerular activation patterns among PNs (Fig. 4c), we asked how these odors are chemically related. We obtained physicochemical properties of these 16 odors (except for geosmin) calculated by the Dragon software with 32 descriptors from [52] and applied cluster analysis based on Euclidean distances among the physicochemical properties of the odors (Additional file 8: Figure S5a, b). Some pairs or combinations of odors that have similar physicochemical properties clustered closely, such as acetophenone and benzaldehyde, 1-octanol, 1-hexanol, and 1-octen-3-ol (Additional file 8: Figure S5a), also clustered in the PN neural space (Fig. 4a). We performed a PCA to visualize the 32-dimensional physicochemical space in three dimensions (Additional file 8: Figure S5c). This map showed similar but slightly modified distribution of the odors in the physicochemical space compared to those in neural space represented by PN odor responses (Fig. 4d and Additional file 8: Figure S5c). These results suggest that PN neural space largely preserves relationships as measured by physicochemical properties of odors. However, the space was not a simple reflection, but was modified. The degree of similarity of the representations was tested by comparing the correlation between physicochemical distances and PN response distances for each odor pair (Additional file 8: Figure S5d). We found a moderate correlation between these two parameters (*r* = 0.518; *p* = 1.43 × 10^–9^). Thus, the physicochemical space is reorganized into PN neural space, where odors are encoded by a combination of AL glomerular groups.

We next addressed the question about how neighboring glomerular groups (defined in Fig. 4c) represent specific odor groups. One hypothesis was that neighboring glomerular groups possess similar odor response profiles because the OSNs projecting to these glomeruli might preserve similar OR protein sequences. Therefore, we examined similarity of OR protein sequences by generating a phylogenetic tree of ORs. The phylogenetic tree of 60 ORs and the distance matrices among these ORs indicated that ORs that project to the same glomerular groups (labeled in the same color) did not cluster but rather spread widely (Additional file 9: Figure S6a, b). Thus, these glomerular groups were not targeted by OSNs expressing similar ORs. Further, the correlation between sequence similarity and glomerular anatomical distances (*r* = 0.168; *p* = 0.00360) and the correlation between sequence similarity and PN response distance (*r* = 0.211; *p* = 0.000246) were very weak (Additional file 9: Figure S6c, d). These results show that neighboring glomerular groups building up the AL functional map are not targeted by OSNs expressing similar OR protein sequences. This relationship can be partly explained by the fact that ORs are so divergent that even within the same species sequence similarity does not necessarily indicate that similar odor response profiles are conserved. Taken together, the similarity of odor response profiles of the PNs innervating the three AL glomerular clusters can thus not simply be explained by OR protein sequence similarity but might be due to other, so far unknown, factors.

### Conversion of the odor representation map from the AL to higher brain centers

How are the odor representations in the AL transferred to higher brain centers, where both convergence and divergence might take place? Jefferis et al. reconstructed the virtual odor response map by combining an anatomical map of PN axonal projections with OSN odor responses [11]. However, no study so far has combined an anatomical projection map with actual odor responses of PNs to map odor representation in the MB calyx and LH. In the present study, we mapped virtual odor representation at the input level of the LH and MB by using the actual odor responses and morphology obtained from multiple PN classes.

**Fig. 5:**
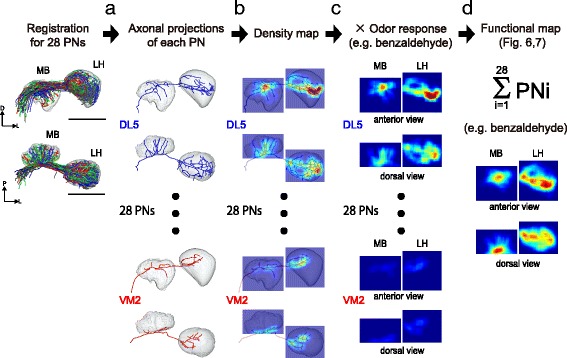
Schematic of reconstructing virtual odor activation maps in the MB calyx and LH. **a** Axons of 28 PN classes were reconstructed and registered to a template MB calyx and LH. **b** Axonal projections were converted to density maps in the MB calyx and LH. **c** Density maps were multiplied by the odor response intensity for each odor to generate response maps for each PN. The response to benzaldehyde is shown as a representative example. **d** The response maps of each PN were summed to generate functional maps in the MB calyx and LH. All calculations were performed for all three dimensions, while the anterior-posterior axis (*top*) and the dorsal-ventral axis (*bottom*) are used for visualization

First, we traced the pathway from each glomerulus by reconstructing axonal projections of its PNs. Among the 67 uniglomerular PNs, axons of 51 PNs were successfully reconstructed in the MB calyx and LH (covering 28 PN classes out of the 31 PN classes from which we recorded). PNs innervating the three glomeruli VA2, VA3, and VM3 could not be reconstructed due to weak staining. In the remaining neurons we reconstructed the axonal projections of each single PN and segmented the MB calyx and LH neuropil in each brain for the 51 PNs. We next applied image registration in order to evaluate the spatial overlap or separation of PNs in the MB calyx and the LH by registering the MB calyx and LH label to a template brain using non-linear surface-matching methods. In order to compare PNs from different animals, the neuron channels were spatially aligned afterwards according to the transformation value calculated by the registration (for details see Methods). We then obtained representative data for each of the 28 glomeruli and generated density maps for these PNs (Fig. 5b, Additional file 10: Figure S7). Before reconstructing the functional maps, we checked how AL odor representation is anatomically wired to higher brain centers. For this purpose, we followed the projections from the three glomerular clusters found in Fig. 4c into the LH and MB calyx and analyzed the similarity of their projection patterns with a hierarchical cluster analysis (see Methods, Additional file 10: Figures S7, Additional file 11: Figure S8). We found no clear segregation among projections from these three glomerular groups in the LH and MB calyx (Additional file 11: Figure S8a, b). There was a weak tendency that the PN classes contributing to representation of attractive odors in the first cluster of Fig. 4a (colored in red) innervate the posterior-dorsal region of the LH (except for the VM7d PN), while the PN classes in the second and third clusters (colored in blue and green respectively) mainly innervated the more ventral region with smaller bifurcated branchings in the medio-dorsal region in the LH (Additional file 10: Figure S7, Additional file 11: Figure S8). As we failed to reconstruct the two PN classes from the first cluster glomerular groups (VA2 and VM3), we compared with VA2 and VM3 PN projection patterns established in the previous study [11] and confirmed that these PN classes project to the posterior-dorsal region of the LH, supporting this tendency. Although the dataset is limited, these results suggest that there might be a loosely hard-wired pathway from the glomerular groups in the AL to the LH, while the AL map is represented in a more distributed fashion in the calyx of the MB.

### Odor valence segregation and integration in the MB and LH

Next, we weighted our measured odor response intensities on the density maps of axonal projections of the 28 PNs (Fig. 5c). Then, we summed odor responses from the 28 PN classes and generated a virtual activity map for each odor (Fig. 5d).

**Fig. 6:**
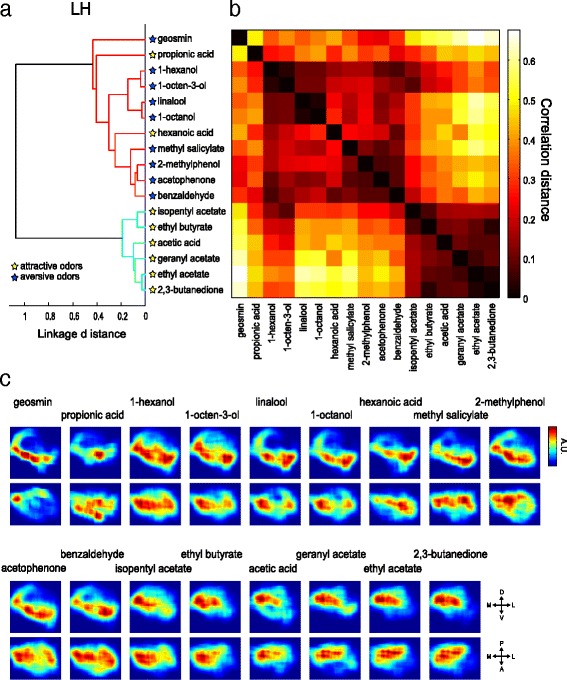
Attractive and aversive odor representations are segregated into two different regions in the LH. **a** Hierarchical cluster analysis for 17 odors based on the correlation distances between the functional maps. The cut-off threshold is set at 0.6 linkage distance, detecting two groups of separately clustered odors colored in *red* and *cyan*. **b** A complete correlation matrix for 17 odors based on the similarities of functional maps in the LH between each odor. Each axis of the matrix is ordered as in **a**. **c** The virtually reconstructed functional maps in the LH are visualized two-dimensionally in the anterior view (*top*) and the horizontal view (*bottom*). Color map is scaled for each image to represent the maximum range

**Fig. 7:**
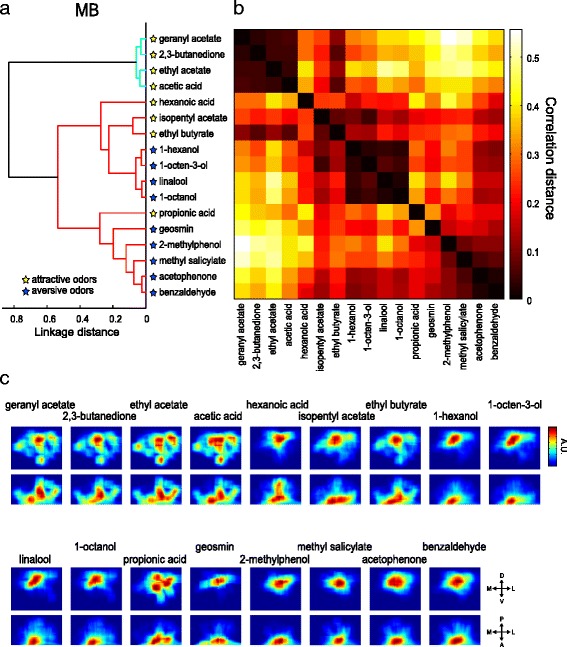
Attractive and aversive odor representations are partly separated in the MB calyx. **a** Hierarchical cluster analysis for 17 odors based on the correlation distances between the functional maps. The cut-off threshold is set at 0.6 linkage distance, detecting two groups of separately clustered odors colored in *red* and *cyan*. **b** A complete correlation matrix for 17 odors based on the similarities of functional maps in the MB calyx between each odor. Each axis of the matrix is ordered as in **a**. **c** The virtually reconstructed functional maps in the MB calyx are visualized two-dimensionally in the anterior view (*top*) and the horizontal view (*bottom*). Color map is scaled for each image to represent the maximum range

Virtually reconstructed summed responses of the 28 PN classes for each odor are shown in Fig. 6 (in the LH) and Fig. 7 (in the MB calyx). We applied a hierarchical cluster analysis to evaluate the similarity of these spatial odor response patterns in the LH and the MB calyx respectively, using correlation distances of the reconstructed activity maps between two pairs of odors (Figs. 6a, b, 7a, b). This method was also used to evaluate the similarity of a three-dimensional density map in [11]. The correlation distance, which is insensitive to intensity, was deemed suitable for comparing patterns of regional activities.

Several previous studies suggested that the LH could be directly linked to innate behaviors [13, 18, 20–22]. Thus, reconstructing the odor representation map in the LH is key to understanding how flies perceive odor valence. Our reconstructed functional maps demonstrated that the attractive and aversive odors were classified into two separate clusters in the LH (Fig. 6a, b). Aversive odors activated ventral regions, while attractive odors activated posterior-dorsal regions (Fig. 6c). These results suggest that odor valence is integrated into two separate regions in the LH. The exceptions were hexanoic acid and propionic acid, which had been categorized as attractive odors in behavioral assays [34] but grouped with the aversive odors (Fig. 6a). This is likely due to our under sampling of acid responding glomeruli (ionotropic receptor projected glomeruli [7]) in the present study, as revealed by rather small total activities induced by these two odors (Additional file 5: Figure S2). Our results further showed that the two aversive odor groups separated in the representations in the AL converged to the ventral regions of the LH. This area might thus form an integration site for information brought by these aversive odors.

The MB, on the other hand, plays a role in circuit plasticity and is involved in learning and memory. Several studies have indicated that the MB is dispensable for innate odor-guided behavior [18–21]. The reconstructed activity maps of the MB calyx revealed that the odors were classified into two clusters; one including exclusively attractive odors and the other including both attractive and aversive odors (Fig. 7a, b). In the MB, attractive odors in the first cluster activated the entire calyx regions including ventral portions, while aversive odors in the second cluster activated more focal regions near the base of the calyx (Fig. 7c). Interestingly, a gradual transition was observed from broad to focal activation patterns in the second cluster, corresponding to an attractive-to-aversive transition. These results suggest that there might be a concentric organization, where the peripheral region of the calyx contributes to attractive behavior while the focal region contributes to aversive, and thereby also suggest that the MB might play an auxiliary role underlying innate odor-guided behavior.

Finally, to address the question of whether labeled line pathways and combinatorial pathways that carry information of similar valence converge in higher brain centers, we focused on the aversive pathway, as our coverage of attractive odor processing channels was less comprehensive than that of aversive ones. Geosmin is processed via a dedicated pathway through the DA2 glomerulus, while eight other aversive odors (benzaldehyde, acetophenone, methyl salicylate, 2-methylphenol, 1-octanol, linalool, 1-octen-3-ol, and 1-hexanol) are represented by combinatorial activation of glomeruli [32, 34] (Fig. 2a, Additional file 5: Figure S2a). In the LH, the eight aversive odors activated the ventral part, while geosmin activated a ventral-posterior region. Although the regions activated by the eight odors extended to the entire region of the ventral LH, there was an overlap at the ventral-posterior region (Fig. 6c). This suggests that the activation patterns of these two different types of aversive odors partially overlap in the LH. In the MB, the geosmin activation pattern was more similar to other aversive odors (Fig. 7c). Our results thus show that the geosmin-activated regions in the LH and the MB calyx are not isolated, but overlap to some degree with regions activated by other aversive odors (Figs. 6c, 7c).

### Temporal dynamics of odor representation

Although previous studies demonstrated that not only spatial but also temporal patterns of the neural code are essential for fine odor discrimination [40], we so far employed only spike numbers during 1-s odor stimuli as an indicator of odor response intensity. Now, we asked whether temporal patterns of PN responses add additional coding capacity to the system under study or not. First, we examined how odor representations evolve in the ensemble PN population over the course of the odor presentation by virtually assembling 31 PN class responses to 17 odors. We described trajectories of three representative odors (ethyl butyrate, acetophenone, and 1-octen-3-ol) by reconstructing PN population responses and visualizing them in a three-dimensional PC space (Additional file 12: Figure S9a). The trajectories showed that odor representations developed rapidly to reach a peak firing rate at 150 ms and slowly returned to the baseline. Next, we reconstructed trajectories for all odors in a two-dimensional PC space. Odors were rapidly separated with a similar pattern as observed by the clustering in Fig. 4a (Additional file 12: Figure S9b). Inter-odor distances between all odor pairs showed that separation peaked at 150 ms and gradually declined to the baseline at 2 s after the onset of odor stimulation. (Additional file 12: Figure S9c). Since previous studies have revealed that slow temporal patterns through de-correlation of mitral cells ensemble activities represent the key factor for fine odor discrimination (e.g., [53]), we examined developing changes of distance matrices during the course of olfactory stimulation. Distance matrices retained a similar degree of distinction during the 1-s odor stimulus, and the pattern disappeared at 1 s after the odor offset (Additional file 12: Figure S9d). We also confirmed similar time course changes in the distance matrices mapped on the LH and MB (Additional file 12: Figure S9e, f). Taken together, these results suggest that in the *Drosophila* AL, slow temporal patterns may not contribute to fine odor discrimination through de-correlation ensemble representation, as has been shown in the zebrafish olfactory system [53]. Therefore, analyzing response intensity during 1-s odor stimulation was a suitable compromise to evaluate odor representation without losing critical information for fine odor discrimination. However, note that we could not address another important temporal factor, “oscillatory synchrony,” which was considered less prominent compared to other olfactory systems but demonstrated in a previous study in the *Drosophila* AL [54], as precise synchronization must be analyzed by local field potentials (LFPs) being recorded simultaneously as a reference.

## Discussion

We used in vivo whole cell patch-clamp recordings and stainings, mapped odor response profiles of 31 glomerular channels, and followed the flow of information into higher brain centers. We characterized odor response profiles of OSNs and PNs in ~30 glomeruli and found that the majority of odors are indeed represented in multiple glomeruli in a combinatorial manner (Fig. 2). Odor representation is thus largely combinatorial, while a few single odors highly important for survival and/or reproduction are processed through dedicated pathways. This organization enables the fly to discriminate a vast number of different odors, while maintaining a very robust and specific odor processing system for a few crucial, directly fitness-related odors. Our results show a rather conservative transformation between OSNs and PNs (Fig. 2) [55, 56]. Earlier studies focused on a few specific glomeruli and generalized the results to the entire system [44, 45]. Our study expands the scale to more than half of the glomerular channels and demonstrates that transformation of odor tuning curves is not homogeneous but glomerulus-dependent (Fig. 3).

Although our PN samplings were comprehensive, covering ~60% of glomerular channels, the depth of replication (the number of samples for each glomerulus) was moderate, as it was limited by technical difficulties. Therefore, inter-individual variation that may exist both in physiological odor responses (Additional file 2: Figure S1) and morphological axonal projection (Additional file 10: Figure S7) should be taken into account. However, our efforts to minimize such biases by the selection of analytical methods and application of registration techniques minimize ambiguity.

Whether a topographical representation that reflects the feature of olfactory stimuli is present is a central issue for olfactory coding. Interestingly, we found a few glomerular groups that are relevant for odor valence representation, often comprising neighboring glomeruli (Fig. 4). This organization tempts us to postulate that through evolution these glomeruli have divided from the same ancestral glomerulus, or one glomerulus has derived from the neighboring glomerulus. However, we found no evidence that these neighboring glomeruli are targeted by OSNs expressing phylogenetically related ORs (Additional file 8: Figure S5). Future studies should address the developmental mechanisms by which a glomerulus can be divided, which might provide an explanation as to why the AL has a topographical map.

Odor valence thus seems to be represented by different clusters of glomeruli in the AL. How is this information transformed into higher brain centers? When we reconstructed a functional map combining the anatomical map with odor response characteristics, we discovered segregated odor valence representation in the LH and partly in the MB calyx.

Several studies have proposed that the LH is the center underlying innate olfactory-dependent behavior [13, 18, 20–22]. Thus, it is postulated that the LH consists of a topographic map where each subregion is dedicated to a different type of innate behavior. An obvious example is found in pheromone channels that project separately from general odor channels in the LH (lateral protocerebrum in other insects) [11, 57, 58]. In *Drosophila*, dedicated pathways processing pheromone-related odors have been studied in detail [27, 28, 59–62]. Other than pheromones, dedicated pathways processing aversive CO_2_ or acids via glomeruli V and DC4 respectively have been shown. These two pathways converge to the medial side of the LH, while information regarding ammonia and amines that are attractive to flies is processed via the VM1 glomerulus, for which PNs converge in the lateral posterior part of the LH [22]. Our group recently showed that dedicated pathways processing aversive geosmin and parasitoid odor information via the DA2 and DL4 glomeruli respectively converge onto very similar regions in the LH [31]. These odors are ecologically important for flies and directly trigger specific behaviors via dedicated neural pathways.

On the other hand, it is largely unknown how more general odors that induce innate odor-guided behavior are represented in the LH. A recent study revealed a topographical LH map relating to hedonic odor valence by imaging inhibitory PNs and ventro-lateral protocerebrum (vlpr) neurons (third order neurons projecting to the vlpr from the LH) [13]. However, how odor information carried by excitatory PNs is represented in the LH has remained unknown. Our results for the first time show that information conveyed by excitatory PNs converges into two regions that could be integration sites for attractive and aversive odors. Attractive odor stimulation activated the posterior-dorsal region of the LH (Fig. 6). This region appears to correspond to the fruit odor processing region shown in [11]. Conversely, aversive odors activated the ventral regions of the LH (Fig. 6). We found that two categories of aversive odors, represented in different clusters of AL glomeruli (Fig. 4a, c), activated largely overlapping regions in the ventral part of the LH (Fig. 6c). Furthermore, the labeled line pathway mediating geosmin aversive information also activated the ventral region of the LH, showing a partial overlap with the activation pattern for other aversive odors that are represented by combinatorial activation of glomeruli. Third order neurons that convey information from this region to the vlpr were also demonstrated in recent studies [12, 13]. In another study, third order neurons conveying attractive information from the dorsal region of the LH to the superior medial protocerebrum were found [63].

The MB consists of numerous Kenyon cells (KCs), among which combinatorial activation patterns of PNs are converted into a sparse representation [64, 65]. Since the connection patterns between PNs and KCs are likely to be random, there would be no hard-wired circuit connecting specific glomeruli to specific KCs [16, 66]. However, a recent study suggests that a KC receives inputs from several different glomeruli and integrates activation evoked by similar odors [67]. In addition, a zonal organization has been found in the MB. The different zones are innervated by different types of KCs, and corresponding coarse regional PN projection patterns were also revealed [15, 17]. Our results revealed that odor valence segregation is partly observed also in the MB calyx (Fig. 7). The role of the MB for innate odor preference is less understood compared to its well-established role for olfactory learning. A previous study demonstrated that the MB is relevant for innate odor attraction, while it is irrelevant for innate odor aversion [18, 68]. Our results suggest that the MB is also able to partly contribute to categorize hedonic odor valence via a concentric organization. Such an organization in the calyx of the MB has already been observed at neuroanatomical levels, where it is proposed that the termination zones schematized as concentric circles from the inner to outer MB calyx correspond to a progression from the anterior-ventral to posterior-dorsal LH [11, 15]. Recent studies demonstrated that ensemble activity of MB output neurons can determine odor-guided behavior and that response profiles of these neurons are individually modulated, possibly by experience [69–71]. It is not clear how the odor map revealed in the MB calyx is read out by different types of KCs and then by the MB output neurons, but the MB output should be important to modulate innate odor-guided behavior by associative learning. Taken together, our results suggest that there is an organization in the LH and MB that allows the insect to categorize odor valence information. Beyond these anatomically defined areas, positive and negative odor information has to be integrated and put into the context of other sensory input and of the physiological state of the insect. This integration can then form the basis for a behavioral decision.

**Fig. 8:**
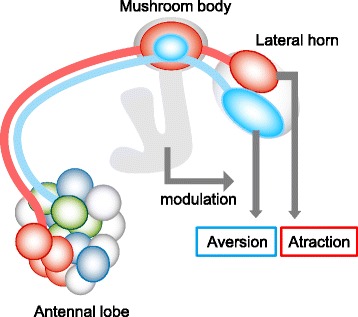
Schematic diagram of the model proposed by our study. Odors are separately represented with three different glomerular groups in the AL, reflecting odor valence as well as chemical structures. Attractive and aversive odors are further segregated and integrated into two different regions in the LH and partly separated in the MB. Innate odor-guided behaviors could be induced by reading out the activation patterns of these two regions in the LH. The MB output could modulate the innate behavior by associative learning (see Discussion)

In the present study, we used the same concentration of each odor (10^–3^ v/v, except for geosmin 10^–4^). How does a concentration change affect our conclusions? In general, higher concentrations of odors activate more receptors, and thereby more glomeruli and PN classes. Also, olfactory researchers empirically know that higher concentration of odor often induces aversive behavior in the fly. From our results and previous findings, we hypothesize that the activation patterns in the LH could be the key determinant for the fly to decide whether it approaches or avoids an odor (Fig. 8). In this scenario an attractive odor activates mainly the posterior-dorsal region of the LH at a certain concentration range, while higher concentration of the odor activates more glomeruli and the activated region spreads into the ventral side of the LH, thereby triggering aversive behavior. Since the opposite (higher concentration of aversive odor turning into attractive) does not happen, the aversive pathway would be dominant to the attractive one. In addition, we only deal with innate behavior. Most odors could be changed in their valence when associated with reward or punishment [19]. Even if odor representation patterns at the PN level do not change, flies can display an opposite behavior. This could be explained by the role of the MB. MB output neurons could overwrite the read-out from the LH [69]. Although our results reveal a snapshot of activation patterns at a single concentration and innate state, they provide the basis for the circuit design underlying innate odor-guided behavior. Further studies will show how these basic olfactory circuits are modulated by experience, physiological state, and multimodal sensory input.

## Conclusions

A fundamental question in olfactory coding is to understand how odor information initially separated by multiple receptor-glomerulus channels is integrated and read out by higher brain centers. In addition, little is known about how odor maps at higher processing centers are related to the animal's perception and innate behavior. The present study provides a major breakthrough to answer these questions, by mapping the olfactory information flow from the periphery to higher brain centers in unprecedented detail, using the *Drosophila* olfactory system. We found that attractive and aversive odors were separately represented by different clusters of glomeruli in the AL, and these representations were segregated into two different regions in the LH and partly separated in the MB. Since it has been increasingly evident that the anatomical organization of the olfactory system in mice has a similar architecture to that of the *Drosophila* olfactory system, our results may provide an important stepping stone toward understanding the common principle of the olfactory circuit design underlying innate olfactory behaviors.

## Methods

### Fly stocks

Flies were maintained on conventional medium under a 12-h light/12-h dark cycle at 25 °C. Adult females 1–3 days after eclosion were used in all experiments. Fly lines used in this study were Canton-S (*n* = 42), GAL4-GH146/UAS-mCD8GFP (*n* = 25), GAL4-NP5221/UAS-mCD8GFP (*n* = 3), and GAL4-NP7217/UAS-mCD8GFP (*n* = 1).

### In vivo whole cell patch-clamp recording

Patch-clamp recordings were performed with the same configuration as previously described (Seki et al. 2010) [72], except that an in vivo preparation was used and odor stimuli were given (Fig. 1b). The in vivo preparation was similar to the one used in imaging experiments (Stökl et al. 2010) [33]. Briefly, flies were anesthetized on ice and fixed in a Plexiglas stage using a copper plate (G220-5, Athene Grids). The neck was fixed with a minutien pin (#26002-10, Fine Science Tools, Foster City, CA, USA), and the back of the head was glued with colophony resin (Royal Oak Rosinio, Royal Oak, Germany) to prevent moving of the head. The antennae were pulled forward with a fine metal wire (Rediohm-800, HP Reid Co., Palm Coast, FL, USA). After a few hours, plastic cover glass (L4193, Plano-em, Germany) with a hole covered by stretched parafilm was put on the head. The parafilm was ripped to expose the surface of the head, and the gap between the parafilm and the head capsule was filled with two component silicone (KWIK-SIL, World Precision Instruments, Sarasota, FL, USA). Then the head capsule was removed to expose the brain. At the same time, the brain was immersed with ringer solution containing (in millimoles) 130 NaCl, 5 KCl, 2 MgCl_2_, 2 CaCl_2_, 36 sucrose, and 5 4-(2-hydroxyethyl)-1-piperazineethanesulfonic acid (HEPES); pH 7.3. Tracheae and muscles were removed for the both AL to be exposed, and the neurolemma was carefully removed with fine forceps. Cell bodies of PNs were visualized under an infrared DIC microscope (BX51WI Olympus, Hamburg, Germany) (Fig. 1c). Whole cell recording was made from a cell body of PN with a glass pipette containing the internal solution: (in millimoles) 140 potassium aspartate, 10 HEPES, 1 KCl, 4 Mg-ATP, 0.5 Na_3_GTP, 1 ethylene glycol-*bis*(β-aminoethyl ether)-*N*,*N*,*N*',*N*'-tetraacetic acid (EGTA), and 1 Lucifer yellow CH or 7 biocytin; pH 7.3. The membrane potential was recorded in current clamp mode using an EPC 10 patch-clamp amplifier (HEKA Elektronik, Lambrecht, Germany) with Patch-master software. The membrane potential was kept around –50 mV when injecting a small current if necessary. One neuron per brain was stimulated, recorded, and stained.

### Odor stimulation

Odorants were diluted (10^–2^ v/v) except for geosmin (mostly 10^–3^, only three PNs were tested with 10^–2^ and they did not respond to 10^–2^ geosmin) in H_2_O (for acetic acid, propionic acid) or mineral oil (for all other odors). Odors used for PN recordings were acetic acid, benzaldehyde, 1-octanol, 2,3-butanedione, linalool, acetophenone, 1-octen-3-ol, ethyl butyrate, methyl salicylate, isopentyl acetate, hexanoic acid, 2-methylphenol, ethyl acetate, geranyl acetate, 1-hexanol, propionic acid, and geosmin. We tested all PNs with all odors and three controls (mineral oil, H_2_O, empty pipette), “20 stimuli as one set of stimuli,” at least one time with the fixed order as written above (i.e., acetic acid, benzaldehyde … geosmin, mineral oil, H_2_O, empty pipette) and proceeded the second round of stimuli until the recording stopped. If we lost recording without completing the whole odor set, we did not use those data for analysis and did not try recording another PN in the same preparation. The stimulus duration was 1 s, and the inter-stimulus interval was ~40 s. 10 μl of the diluted odors was put on the filter paper (Whatman), placed inside a glass Pasteur pipette. A stimulus controller (Stimulus Controller CS-55, Syntech, Kirchzarten, Germany) was used to produce a continuous airstream (1.2 L/min), which was joined with the airstream (0.12 L/min) from an empty pipette at 8 cm from the end of the delivery tube, and to switch to the stream from an odor stimulus pipette (0.12 L/min), which was joined to the continuous airstream at 6.5 cm from the end of the delivery tube. The end of the delivery tube, which was 5 mm in diameter, was placed 8 mm from the fly. The applied odor stimuli were thus additionally diluted approximately 10 times when merging to the continuous airstream.

### Single sensillum recording (SSR)

SSR measurements were performed as described previously [32]. The recording electrode and the reference electrode (inserted into the eye) were positioned under a microscope (Olympus BX51W1). The recording electrode was positioned by using a motorized, piezo-translator-equipped micromanipulator (Märzhäuser DC-3 K/PM-10, Wetzlar-Steindorf, Germany). The signal was amplified (Syntech UN-06), digitally converted (Syntech IDAC-4), and finally visualized and analyzed by using Syntech AutoSpike v3.2.

### Morphological analysis

To visualize glomerular structures and regions in the higher brain centers, nc82 immunostainings were performed as previously described (Seki et al. 2010) [72]. Additionally, biocytin-injected neurons were labeled with (1:500) Alexa Fluor 555 streptavidin (S-32355, Invitrogen), which was incubated with the secondary antibody for nc82, (1:200) goat anti-mouse Alexa Fluor 633 (A21052, Invitrogen). Images were taken with a Zeiss LSM510 META confocal microscope (Carl Zeiss, Jena, Germany). Axons of the stained PNs were reconstructed using the Amira 4.1.1 or 5.3.3 software (Visage Imaging, Berlin, Germany) as previously described [72]. For neuron reconstruction the Skeleton plugin [73] of Amira was used. This module allowed the tracing process and created vertex models in which neurites were approximated by cylinders of a particular diameter. The innervated glomeruli were identified based on the glomerular map in [5], except that the “Vm7” and the unnamed “1” glomerulus in [5] were called “Vm7d” and “Vm7v” respectively, according to the recent nomenclature [14].

### Registration method for PN axonal projection

Axonal projections of a single PN were reconstructed, and the MB calyx and LH structures were segmented in each brain. The MB calyx and LH were registered to a template brain using non-linear surface-matching methods. Image stacks were imported to Amira 5.6 (Fei), and neuronal arborizations and neuropils were manually segmented. For registration of datasets a label template of the central brain (MB calyx and LH) was chosen out of *n* = 58 preparations as described in [74]. The warping of segmented labels of the MB calyx and LH onto the template was done in a two-step process: an affine transformation with 12 degrees of freedom (DOF) followed by an elastic registration using modules of Amira. The calculated transformation matrix was applied to the neuron reconstructions that were thus transformed to the template reference space.

### Data analysis

#### Response intensity

PN responses were evaluated using spike numbers during the 1-s odor stimulus (0.05 s after the onset of stimuli to 1.05 s; 0.05 s was an approximate delay for the odors to reach the antennae and pulps from the onset of the stimulus pulse). Spike numbers used for analysis were made by subtraction with the control stimulus (either mineral oil or H_2_O, depending on the solvent for the odors). The response amplitude (spike numbers/s) was calculated using the responses in the first round of stimuli to achieve precise comparison among different PNs under virtually the exact same condition. The mean response amplitude was calculated if more than two PNs were recorded from the same glomerulus. Recording data were analyzed in Igor Pro (Wavemetrics, Lake Oswego, OR, USA) and MATLAB (The MathWorks, Natick, MA, USA) using custom software.

#### Lifetime sparseness

The lifetime sparseness (see Fig. 3) was calculated to quantify the selectivity of a neuron’s odor response profile [45]:

$$ S=\left(\frac{1}{1-\frac{1}{N}}\right)\left(1-{\left({\displaystyle {\sum}_{j=1}^N{r}_j/ N}\right)}^2/\left({\displaystyle {\sum}_{j=1}^N{r}_j^2/\mathrm{N}}\right)\right) $$

where *N* = number of odors (17 in this study), and *r*_*j*_ is the odor response intensity of the neuron to odor *j*. Any values of *r*_*j*_ <0 were set to zero before computing lifetime sparseness.

#### Density map

The density maps of axonal projections of PNs in the MB calyx and LH were generated similarly to the process in [11]. (See Fig. 5, Additional file 11: Figure S8.) Each neuron was divided into segments of length equal to 0.5 μm by setting the interval of vertices as 0.5 μm. To generate a density map in the space of the MB calyx and LH, the voxel size was set to 1 μm^3^. The central points of each segment (vertex) were binned onto the grid of 135 × 135 × 105 voxels. A three-dimensional histogram was generated for each PN axonal innervation in the MB calyx region (55 × 46 × 32 μm) and the LH region (52 × 53 × 43 μm) by counting the vertices in each 1 μm^3^ voxel. Then the histogram was filtered three dimensionally with a boxcar filter (9 pixels). To visualize a two-dimensional projection, each column of voxels was integrated along the projection axis.

#### Functional map

We generated the predicted odor response map as a functional map in the MB calyx and LH. (See Figs. 6 and 7). On the basis of density maps generated for each PN class, the odor response intensity value (any values <0 were set to zero) was multiplied by the density value and then summed with all the PN classes. The similarities of functional maps of each odor were compared by using cluster analysis with correlation distances and Ward’s classification method.

#### Physicochemical analysis

Physicochemical properties of 16 odors calculated with a set of 32 descriptors using Dragon were obtained from [52].

#### Phylogenetic tree and odorant receptor sequence analysis

Sixty aligned OR amino acid sequences of *D. melanogaster* were obtained from the Database of Olfactory Receptors (DOR; http://caps.ncbs.res.in/DOR/index.html) [75]. The distance matrix and the phylogenetic tree were generated using Molecular Evolutionary Genetics Analysis version 7 (MEGA7) [76]. Analyses were conducted using the Jones-Taylor-Thornton (JTT) matrix-based model [77]. The analysis involved 60 amino acid sequences. All positions containing gaps and missing data were eliminated. The evolutionary history was inferred using the neighbor-joining method [78]. The percentage of replicate trees in which the associated ORs clustered together in the bootstrap test (1000 replicates) is shown next to the branches [79]. The tree is drawn to scale, with branch lengths in the same units as those of the evolutionary distances used to infer the phylogenetic tree.

The PCA and hierarchical cluster analysis were performed using MATLAB. Hierarchical cluster analysis was performed using cosine distance (Fig. 4a), Euclidean distance (Additional file 7: Figure S4, Additional file 8: Figure S5, Additional file 9: Figure S6) or correlation distance (Figs. 6, 7 and Additional file 11: Figure S8), and Ward’s classification method. One-way ANOSIM (Bray-Curtis similarity, sequential Bonferroni correction for ties, 10,000 permutations) was done with Paleontological STatistics (PAST) software (https://folk.uio.no/ohammer/past/).

Data are given as “mean ± s.d., n number”, unless otherwise noted.

## Additional files

Additional file 1: Table S1. Odor list. (DOCX 63 kb)

Additional file 2: Figure S1. Odor response intensity of all individual PNs. Odor response intensity of each PN to 17 odors for each glomerulus, calculated by using spike frequencies during a 1-s odor stimulation period. *n* indicates the number of PNs recorded for each glomerulus. Odor responses of different PNs within the same PN class are indicated by different colors. The order of the 17 odors is arranged as in the *inset* on the *bottom right* for all graphs. (PDF 822 kb)

Additional file 3: Table S2. Odor responses of 31 PN classes to 17 odors. (XLSX 18 kb)

Additional file 4: Table S3. Odor responses of 29 OSN classes to 17 odors. (XLSX 17 kb)

Additional file 5: Figure S2. Similar spatial odor representation patterns between PNs and OSNs in the AL. (a) Spatial response patterns for each odor are reconstructed on a template AL using odor response intensity of the 31 PN classes. (b) Spatial response patterns for each odor are reconstructed on a template AL using odor response intensity of the 29 OSN classes. In each map, the AL is viewed from anterior (*top*) and posterior (*bottom*). Each glomerulus name is indicated on the template AL (*bottom right*). Scale bars = 20 μm. (PDF 105472 kb)

Additional file 6: Figure S3. Correlation between glomerular distance and PN odor response similarity. Scatterplot of anatomical glomerular distance versus PN response distance for all 465 pairwise combinations of the 31 glomeruli. Pairwise distance of PN response distance was calculated based on odor response of 31 PN classes using cosine distances for 17 odors. (PDF 740 kb)

Additional file 7: Figure S4. Odor representation analyzed with Euclidean distances. (a) Hierarchical cluster analysis for 17 odors based on the Euclidean distances between odor response intensities of 31 PN classes. The cut-off threshold is set at 70% of the maximum linkage distance, detecting three groups of separately clustered odors colored in *red*, *blue*, and *green*. (b) A complete distance matrix using Euclidean distances for 17 odors based on odor response intensities of the 31 PN classes. Each axis of the matrix is ordered as in (a). (PDF 486 kb)

Additional file 8: Figure S5. Physicochemical properties of odors partly correlate with AL odor representation. (a) Hierarchical cluster analysis for the 16 odors (except for geosmin, due to no data available) based on the Euclidean distances between physicochemical properties of the 16 odors. Glomerular names are colored according to the three clusters found in Fig. 4a, to facilitate comparison between these two analyses. (b) A complete distance matrix measured with Euclidean distances for 16 odors based on physicochemical properties. Each axis of the matrix is ordered as in (a). (c) Principal component analyses for the 16 odors based on physicochemical properties. The percentages of variance accounted by each PC component are shown on each axis. (d) Scatterplot of physicochemical distance versus PN response distance for all 120 pairwise combinations of the 16 odors. Pairwise distance of odors based on PN odor responses was calculated as in Fig. 4b. (PDF 762 kb)

Additional file 9: Figure S6. Sequence similarity of odorant receptors (*ORs*) is not the main factor to determine odor response similarity in the AL glomerular clusters. (a) Phylogenetic tree of *D. melanogaster* 60 ORs. The alignment data of the 60 ORs were obtained from the Database of Olfactory Receptors (*DOR*; http://caps.ncbs.res.in/DOR/index.html), and the phylogenetic tree of the 60 ORs was calculated according to [75]. The percentages of replicate trees in which the associated ORs clustered together in the bootstrap test (1000 replicates) are shown next to the branches. Glomerular name is indicated after the OR name if it is identified in the established OR-glomerulus map [5]. Glomerular names are colored according to the three glomerular clusters detected in Fig. 4c. (b) A complete distance matrix measured with Euclidean distances for 25 OR sequence similarities. (c) Scatterplot of sequence distance versus anatomical glomerular distance for all 300 pairwise combinations of the 25 ORs and the 25 glomeruli. (d) Scatterplot of sequence distance versus PN response distance for all 300 pairwise combinations of the 25 ORs and the 25 glomeruli. Pairwise distance of PN response distance was calculated based on odor response of 25 PN classes using cosine distances for 17 odors. Multiple receptors co-expressed in the same OSN class and project to the same glomerulus (i.e., DL3 and DL4) have been excluded in these analyses (c, d). (PDF 842 kb)

Additional file 10: Figure S7. Axonal projections of PNs in the LH and in the MB. (a) The position of glomeruli are mapped on a template AL. (b) Reconstructed axonal projections in the MB calyx and LH. The PNs are labeled with the same color scheme as in (a). (c) Individual traces of reconstructed axonal projections for the 28 PN classes after registration to the template MB calyx and LH. PNs and glomeruli are colored according to the clusters in Fig. 4c, except for VM2 (*magenta*), which is included in all the three clusters, and D, DM6 (*cyan*), which are included in the second and third clusters. Anterior view (*top*) and dorsal view (*bottom*). Scale bars = 50 μm. (PDF 43110 kb)

Additional file 11: Figure S8. Axonal density maps of PNs in the LH and in the MB. (a) Hierarchical cluster analysis for 28 PN classes based on the correlation distances between axonal density maps in the LH. (b) Hierarchical cluster analysis for 28 PN classes based on the correlation distances between axonal density maps in the MB. (c) The density maps of axonal projections of each PN class are visualized two-dimensionally in the LH. The pseudo color for each PN is scaled respectively. (d) The density maps of axonal projections of each PN class are visualized two-dimensionally in the MB. The pseudo color for each PN is scaled respectively. (PDF 755 kb)

Additional file 12: Figure S9. Temporal dynamics of odor representation. (a) Represented trajectories of PN ensemble activities for three odors (*red*: ethyl butyrate, *blue*: acetophenone, *green*: 1-octen-3-ol) visualized in the three-dimensional PC space. Color is matched with Fig. 4a. Each trajectory is reconstructed with 100-ms steps indicated with *circles,* and *filled circles* indicate 150, 450, and 950-ms time frames. (b) Two-dimensional view of the odor response trajectories for all odors. The odors within the same cluster (colored in *red*, *blue*, *green*, and *gray*) in Fig. 4a have similar trajectories compared to those between different clusters. (c) Inter-odor distances between pairs of odors measured by ensemble PN odor responses with Euclidean distances. (d) Distance matrices of odor representations by PNs at different time frames. Odors are ordered in the same order as in Fig. 4b, to facilitate comparison to the pattern reconstructed with the mean firing rate for 1-s odor stimuli. Clustering of odors remain largely distinct during 1-s odor stimulus but disperse at 2 s. (e), (f) Distance matrices of odor representations in the LH (e) and MB (f) at different time frames. Odors are ordered in the same order as in Fig. 6b (for LH) and Fig. 7b (for MB) to facilitate comparison to the pattern reconstructed with the mean firing rate for 1-s odor stimuli. (PDF 994 kb)

## Abbreviations

AL: Antennal lobe
KC: Kenyon cell
LH: Lateral horn
MB: Mushroom body
OR: Odorant receptor
OSN: Olfactory sensory neuron
PN: Projection neuron
SSR: Single sensillum recording
vlpr: Ventro-lateral protocerebrum
