# Supplementary material for: Olfactory coding from the periphery to higher brain centers in the Drosophila brain
Source: BMC Biol. 2017 Jun 30;15:56. doi: 10.1186/s12915-017-0389-z (PMC5493115; doi:10.1186/s12915-017-0389-z)
Supplement: Supplementary file 1 — Odor list. (DOCX 63 kb) [file 12915_2017_389_MOESM1_ESM.docx]

**Additional file : Table S1. Odor list**

| Odors | CAS No. | MW (g/mol) | Structure | Assigned valence | T-maze^*4)^ | Trap assay^*1)^ | FlyWalk^*2)^ |
| --- | --- | --- | --- | --- | --- | --- | --- |
| acetic acid | 64-19-7 | 60.0 |  | Attractive | ○*4) | ○*1) | N/T |
| benzaldehyde | 100-52-7 | 106.1 |  | Aversive | ●*4) | ●*1) | ●*3)*6) |
| 1-octanol | 111-87-5 | 130.2 |  | Aversive | N/T | ●*1) | ●*6) |
| 2,3-butanedione | 431-03-8 | 86.1 |  | Attractive | ○*4) | ○*1) | ○*3)*6) |
| linalool | 78-70-6 | 154.2 |  | Aversive | ●*4) | ●*1) | ▬*7) |
| acetophenone | 98-86-2 | 120.1 |  | Aversive | ●*4) | ●*1) | ▬*7) |
| 1-octen-3-ol | 3391-86-4 | 128.2 |  | Aversive | ●*4) | ●*1) | ●*3)*6) |
| ethyl butyrate | 105-54-4 | 116.2 |  | Attractive | ○*2) | ▬*1) | ○*6) |
| methyl salicylate | 119-36-8 | 152.1 |  | Aversive | ●*4) | ▬*1) | ●*6) |
| isopentyl acetate | 123-92-2 | 130.2 |  | Attractive | N/T | ▬*1) | ○*6) |
| hexanoic acid | 142-62-1 | 116.2 |  | Attractive | N/T | ○*1) | ○*7) |
| 2-methylphenol | 95-48-7 | 108.1 |  | Aversive | N/T | ●*1) | N/T |
| ethyl acetate | 141-78-6 | 88.1 |  | Attractive | N/T | ▬*1) | ○*3)*6) |
| geranyl acetate | 105-87-3 | 196.3 |  | Attractive | N/T | ○*1) | ○*7) |
| 1-hexanol | 111-27-3 | 102.2 |  | Aversive | ●*5) | ▬*1) | N/T |
| propionic acid | 79-09-4 | 74.1 |  | Attractive | ○*4) | ○*1) | N/T |
| geosmin | 19700-21-1 | 182.3 |  | Aversive | ●*2) | N/T | ●*2) |

○: Attractive ●: Aversive ▬: Neutral N/T: Not tested

17 odors used in this study were assigned as attractive or aversive based on the experiments performed by our group in previous studies [13, 32, 34, 37-39]. If flies showed attractive (or aversive) behavior in at least one of the three assays (the trap assay, T-maze assay and FlyWalk assay), then the odor was defined as attractive (or aversive). The symbols denote an attractive (○), aversive (●) or a neutral (▬) behavioral response. N/T, not tested. The majority of odors yielded similar results independent of the behavioral assay used. In some cases an attractive (or aversive) odor evoked a neutral response (i.e., no response), but contradiction (i.e. attractive in one assay and aversive in another assay) was never observed in the same odor.

*1) Knaden, M., Strutz, A., Ahsan, J., Sachse, S., and Hansson, B.S. (2012). Spatial representation of odorant valence in an insect brain. Cell reports 1, 392-399.

*2) Stensmyr, M.C., Dweck, H.K., Farhan, A., Ibba, I., Strutz, A., Mukunda, L., Linz, J., Grabe, V., Steck, K., Lavista-Llanos, S.*, et al.* (2012). A conserved dedicated olfactory circuit for detecting harmful microbes in Drosophila. Cell 151, 1345-1357.

*3) Steck, K., Veit, D., Grandy, R., Badia, S.B., Mathews, Z., Verschure, P., Hansson, B.S., and Knaden, M. (2012). A high-throughput behavioral paradigm for Drosophila olfaction - The Flywalk. Scientific reports 2, 361.

*4) Strutz, A., Soelter, J., Baschwitz, A., Farhan, A., Grabe, V., Rybak, J., Knaden, M., Schmuker, M., Hansson, B.S., and Sachse, S. (2014). Decoding odor quality and intensity in the Drosophila brain. eLife 3.

*5) Stensmyr, M.C., Giordano, E., Balloi, A., Angioy, A.M., and Hansson, B.S. (2003). Novel natural ligands for Drosophila olfactory receptor neurones. The Journal of experimental biology 206, 715-724.

*6) Thoma, M., Hansson, B.S., and Knaden, M. (2014). Compound valence is conserved in binary odor mixtures in Drosophila melanogaster. The Journal of experimental biology 217, 3645-3655.

*7) pers. comm. M Knaden
