# Supplementary figures and images for: Olfactory coding from the periphery to higher brain centers in the Drosophila brain
Source: BMC Biol. 2017 Jun 30;15:56. doi: 10.1186/s12915-017-0389-z (PMC5493115; doi:10.1186/s12915-017-0389-z)

# Supplementary Figure 1

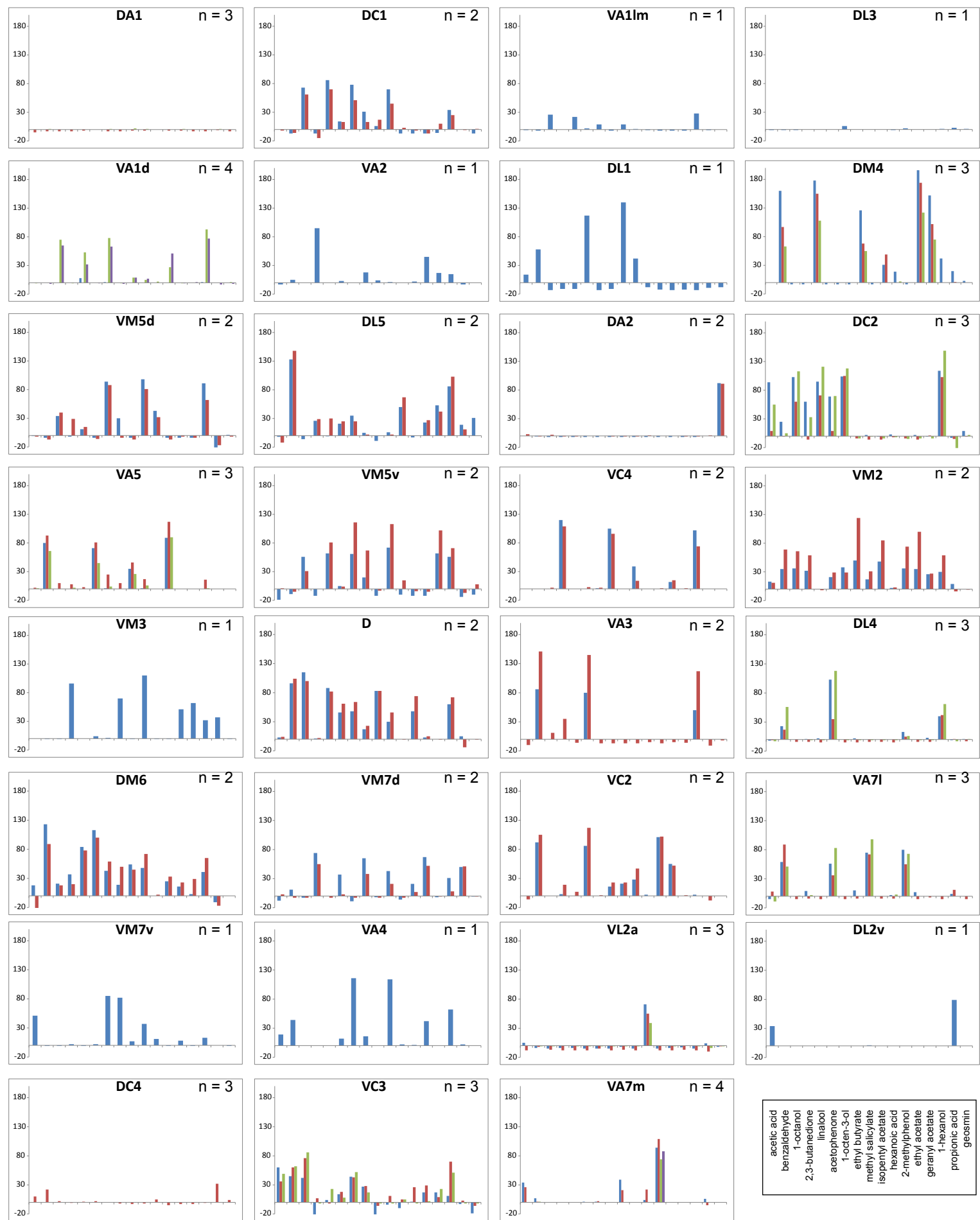

Supplement: Supplementary file 2 — Odor response intensity of all individual PNs. Odor response intensity of each PN to 17 odors for each glomerulus, calculated by using spike frequencies during a 1-s odor stimulation period. n indicates the number of PNs recorded for each glomerulus. Odor responses of different PNs within the same PN class are indicated by different colors. The order of the 17 odors is arranged as in the inset on the bottom right for all graphs. (PDF 822 kb) [file 12915_2017_389_MOESM2_ESM.pdf]

# Supplementary Figure 2

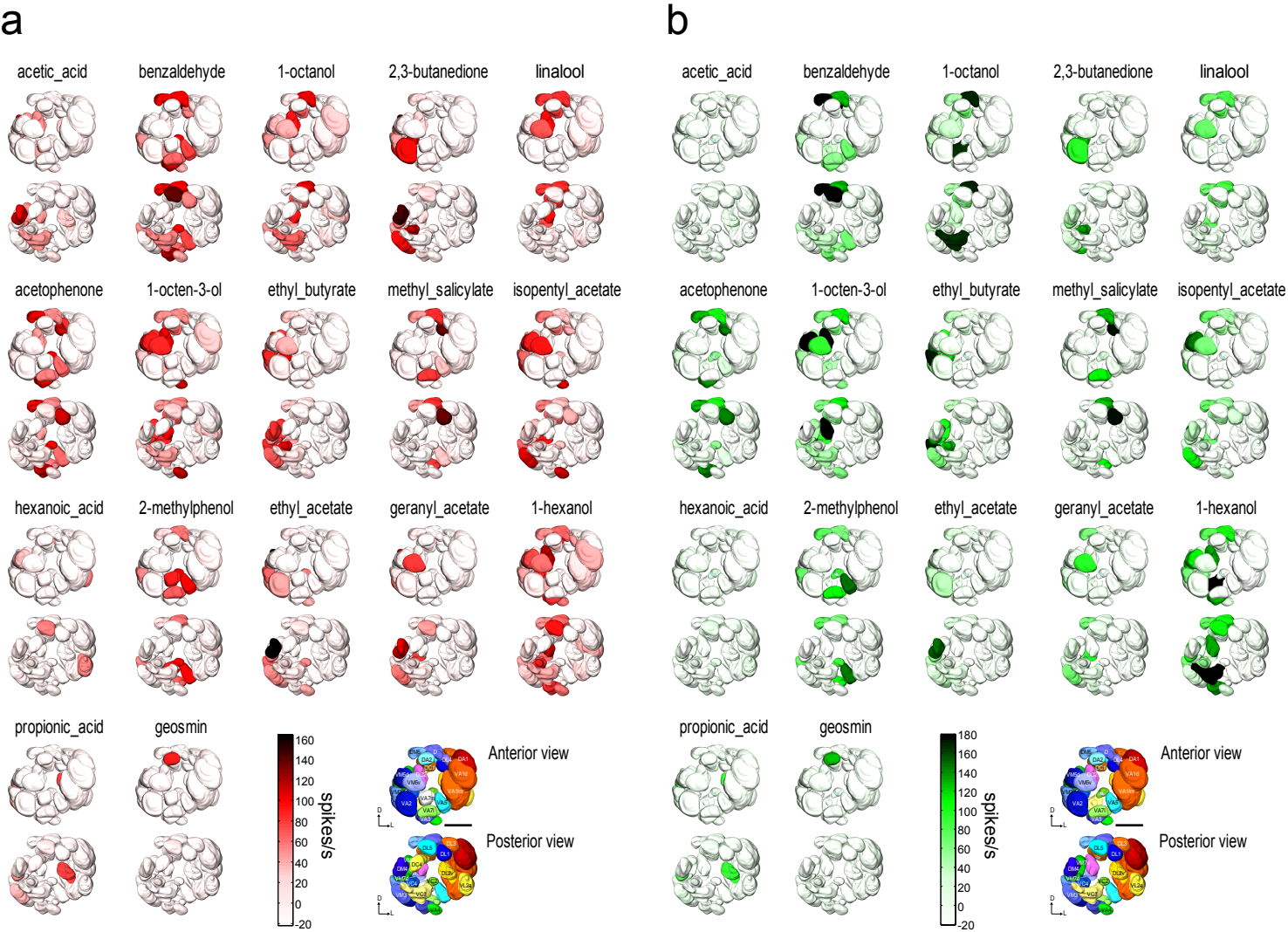

Supplement: Supplementary file 5 — Similar spatial odor representation patterns between PNs and OSNs in the AL. (a) Spatial response patterns for each odor are reconstructed on a template AL using odor response intensity of the 31 PN classes. (b) Spatial response patterns for each odor are reconstructed on a template AL using odor response intensity of the 29 OSN classes. In each map, the AL is viewed from anterior (top) and posterior (bottom). Each glomerulus name is indicated on the template AL (bottom right). Scale bars = 20 μm. (PDF 105472 kb) [file 12915_2017_389_MOESM5_ESM.pdf]

Supplementary Figure 3

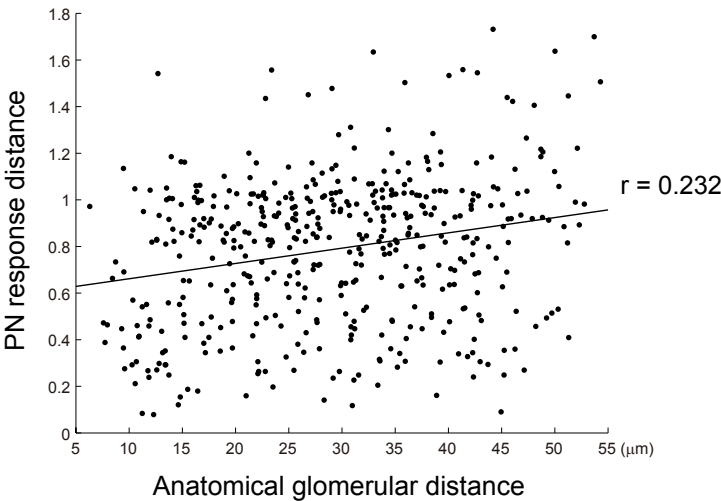

Supplement: Supplementary file 6 — Correlation between glomerular distance and PN odor response similarity. Scatterplot of anatomical glomerular distance versus PN response distance for all 465 pairwise combinations of the 31 glomeruli. Pairwise distance of PN response distance was calculated based on odor response of 31 PN classes using cosine distances for 17 odors. (PDF 740 kb) [file 12915_2017_389_MOESM6_ESM.pdf]

# Supplementary Figure 4

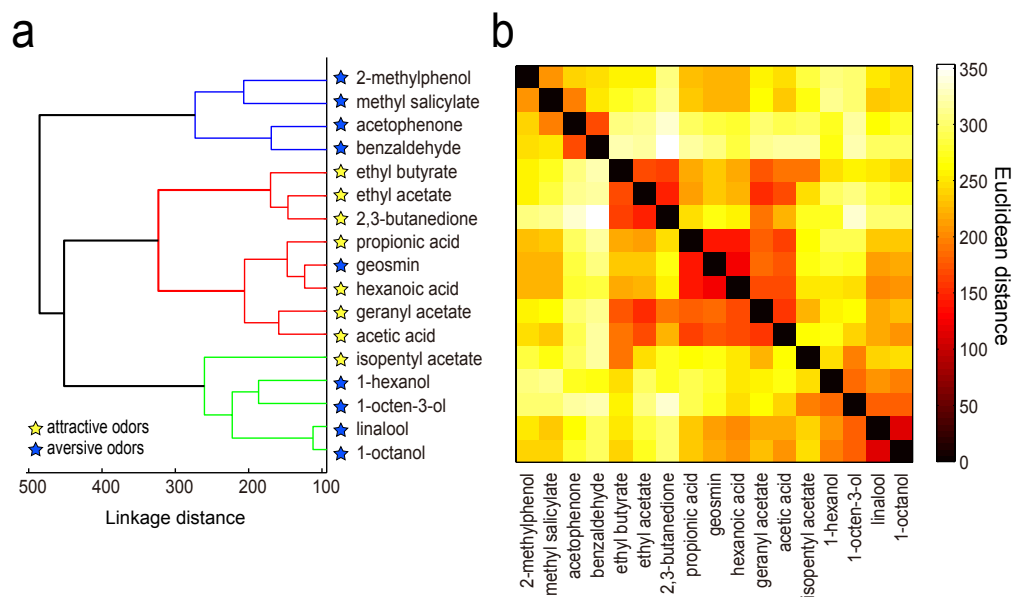

Supplement: Supplementary file 7 — Odor representation analyzed with Euclidean distances. (a) Hierarchical cluster analysis for 17 odors based on the Euclidean distances between odor response intensities of 31 PN classes. The cut-off threshold is set at 70% of the maximum linkage distance, detecting three groups of separately clustered odors colored in red, blue, and green. (b) A complete distance matrix using Euclidean distances for 17 odors based on odor response intensities of the 31 PN classes. Each axis of the matrix is ordered as in (a). (PDF 486 kb) [file 12915_2017_389_MOESM7_ESM.pdf]

# Supplementary Figure 5

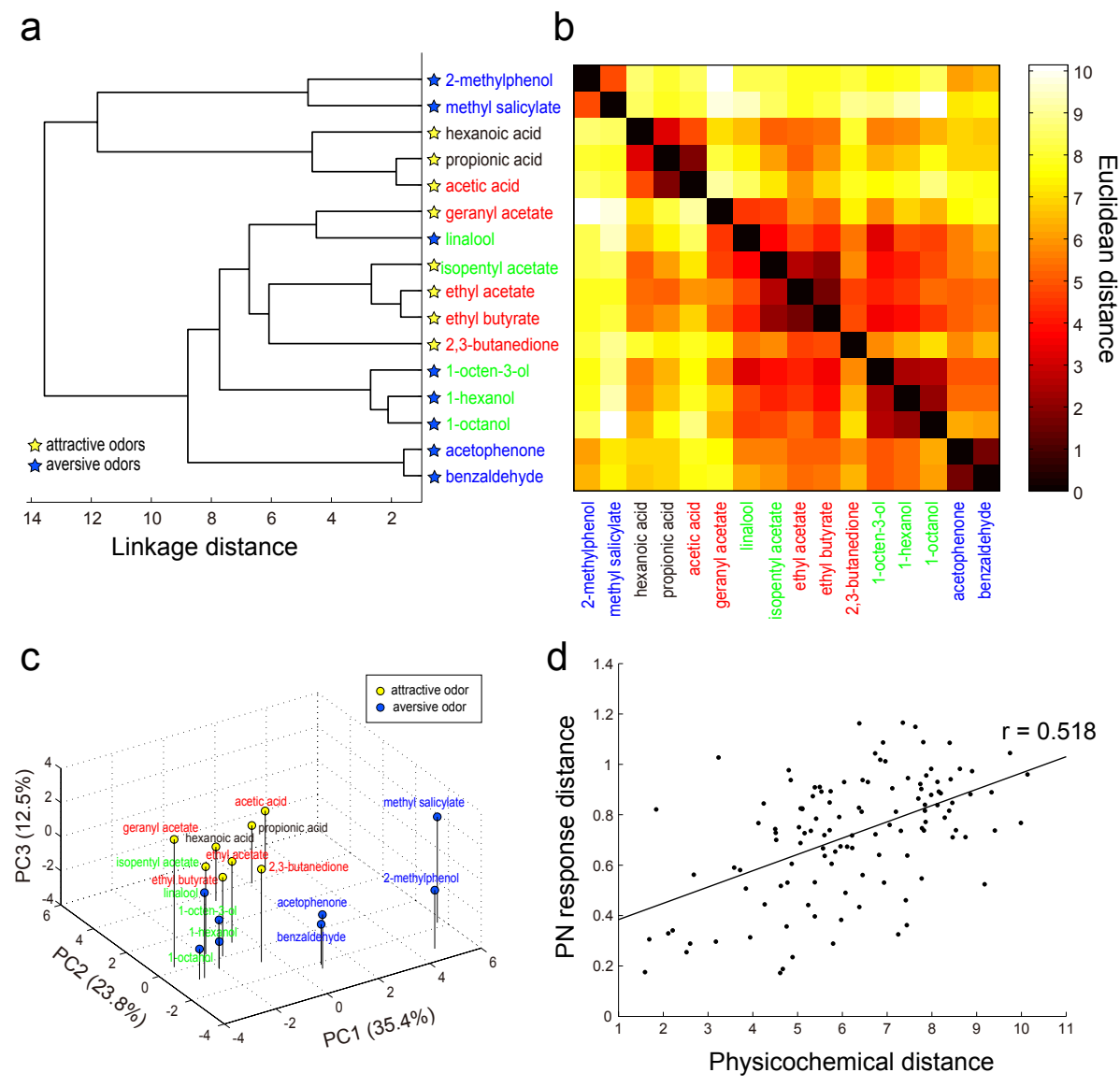

Supplement: Supplementary file 8 — Physicochemical properties of odors partly correlate with AL odor representation. (a) Hierarchical cluster analysis for the 16 odors (except for geosmin, due to no data available) based on the Euclidean distances between physicochemical properties of the 16 odors. Glomerular names are colored according to the three clusters found in Fig. 4a, to facilitate comparison between these two analyses. (b) A complete distance matrix measured with Euclidean distances for 16 odors based on physicochemical properties. Each axis of the matrix is ordered as in (a). (c) Principal component analyses for the 16 odors based on physicochemical properties. The percentages of variance accounted by each PC component are shown on each axis. (d) Scatterplot of physicochemical distance versus PN response distance for all 120 pairwise combinations of the 16 odors. Pairwise distance of odors based on PN odor responses was calculated as in Fig. 4b. (PDF 762 kb) [file 12915_2017_389_MOESM8_ESM.pdf]

# Supplementary Figure 6

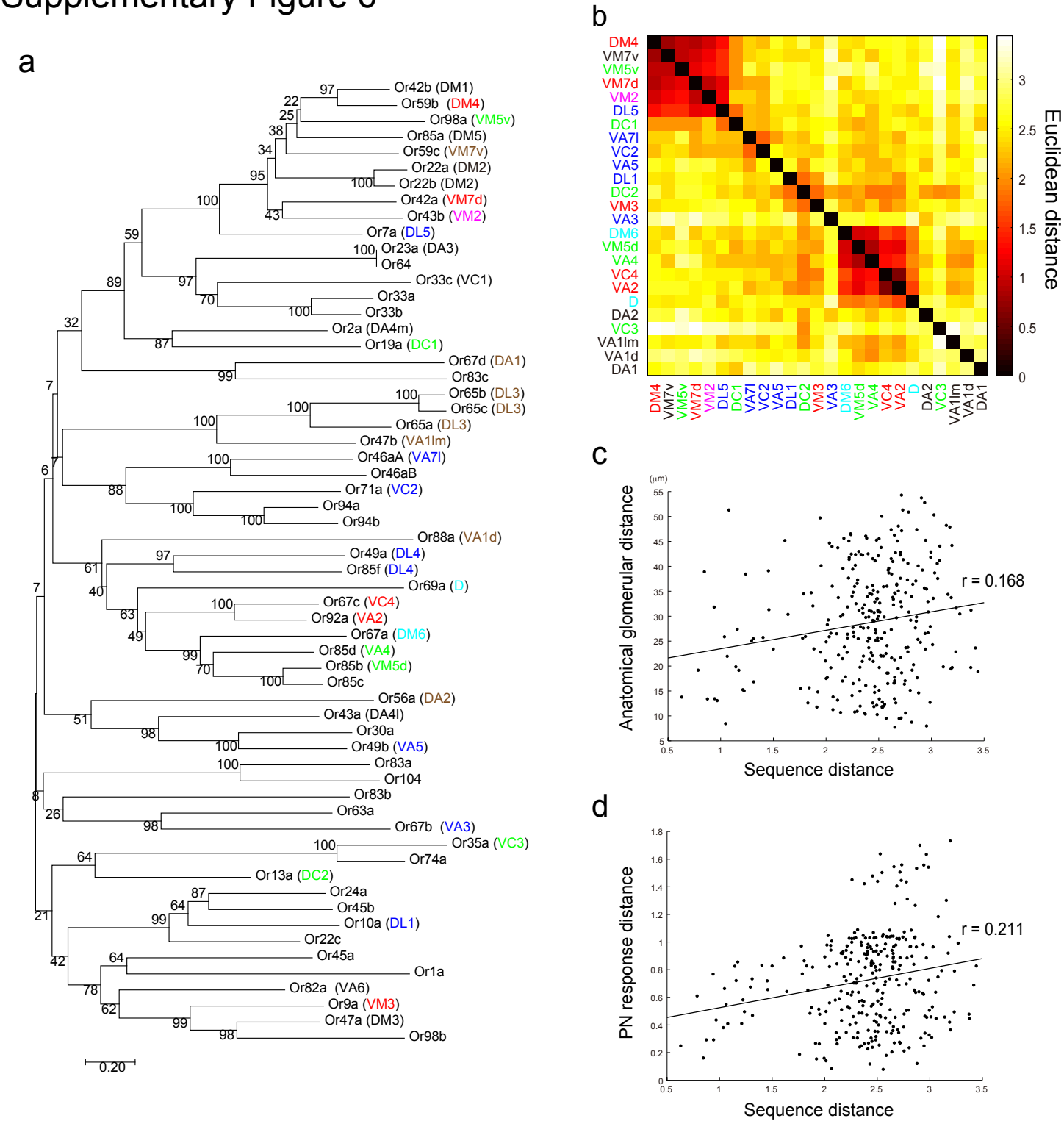

Supplement: Supplementary file 9 — Sequence similarity of odorant receptors (ORs) is not the main factor to determine odor response similarity in the AL glomerular clusters. (a) Phylogenetic tree of D. melanogaster 60 ORs. The alignment data of the 60 ORs were obtained from the Database of Olfactory Receptors (DOR; http://caps.ncbs.res.in/DOR/index.html), and the phylogenetic tree of the 60 ORs was calculated according to [75]. The percentages of replicate trees in which the associated ORs clustered together in the bootstrap test (1000 replicates) are shown next to the branches. Glomerular name is indicated after the OR name if it is identified in the established OR-glomerulus map [5]. Glomerular names are colored according to the three glomerular clusters detected in Fig. 4c. (b) A complete distance matrix measured with Euclidean distances for 25 OR sequence similarities. (c) Scatterplot of sequence distance versus anatomical glomerular distance for all 300 pairwise combinations of the 25 ORs and the 25 glomeruli. (d) Scatterplot of sequence distance versus PN response distance for all 300 pairwise combinations of the 25 ORs and the 25 glomeruli. Pairwise distance of PN response distance was calculated based on odor response of 25 PN classes using cosine distances for 17 odors. Multiple receptors co-expressed in the same OSN class and project to the same glomerulus (i.e., DL3 and DL4) have been excluded in these analyses (c, d). (PDF 842 kb) [file 12915_2017_389_MOESM9_ESM.pdf]

Supplementary Figure 7

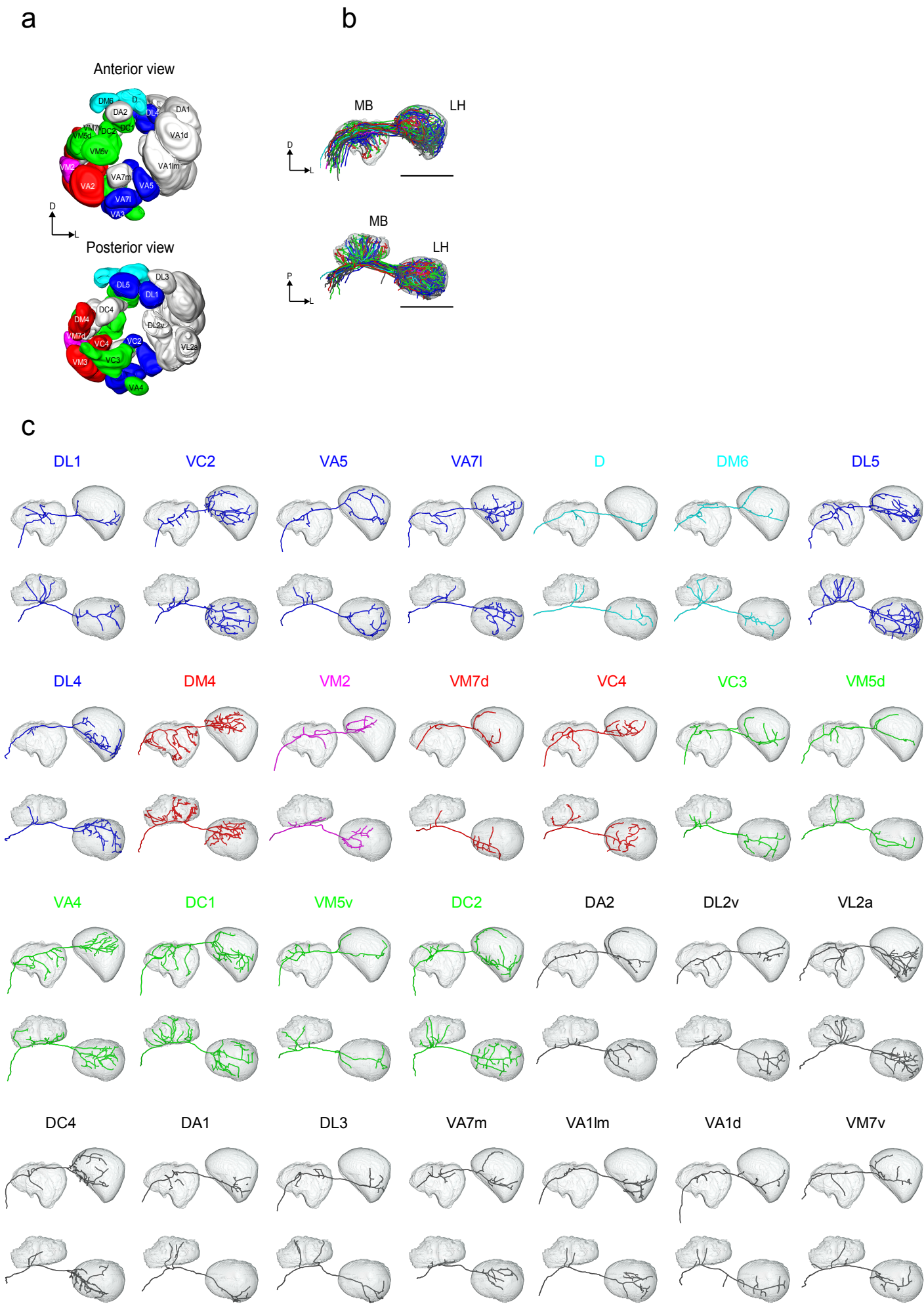

Supplement: Supplementary file 10 — Axonal projections of PNs in the LH and in the MB. (a) The position of glomeruli are mapped on a template AL. (b) Reconstructed axonal projections in the MB calyx and LH. The PNs are labeled with the same color scheme as in (a). (c) Individual traces of reconstructed axonal projections for the 28 PN classes after registration to the template MB calyx and LH. PNs and glomeruli are colored according to the clusters in Fig. 4c, except for VM2 (magenta), which is included in all the three clusters, and D, DM6 (cyan), which are included in the second and third clusters. Anterior view (top) and dorsal view (bottom). Scale bars = 50 μm. (PDF 43110 kb) [file 12915_2017_389_MOESM10_ESM.pdf]

Supplementary Figure 8

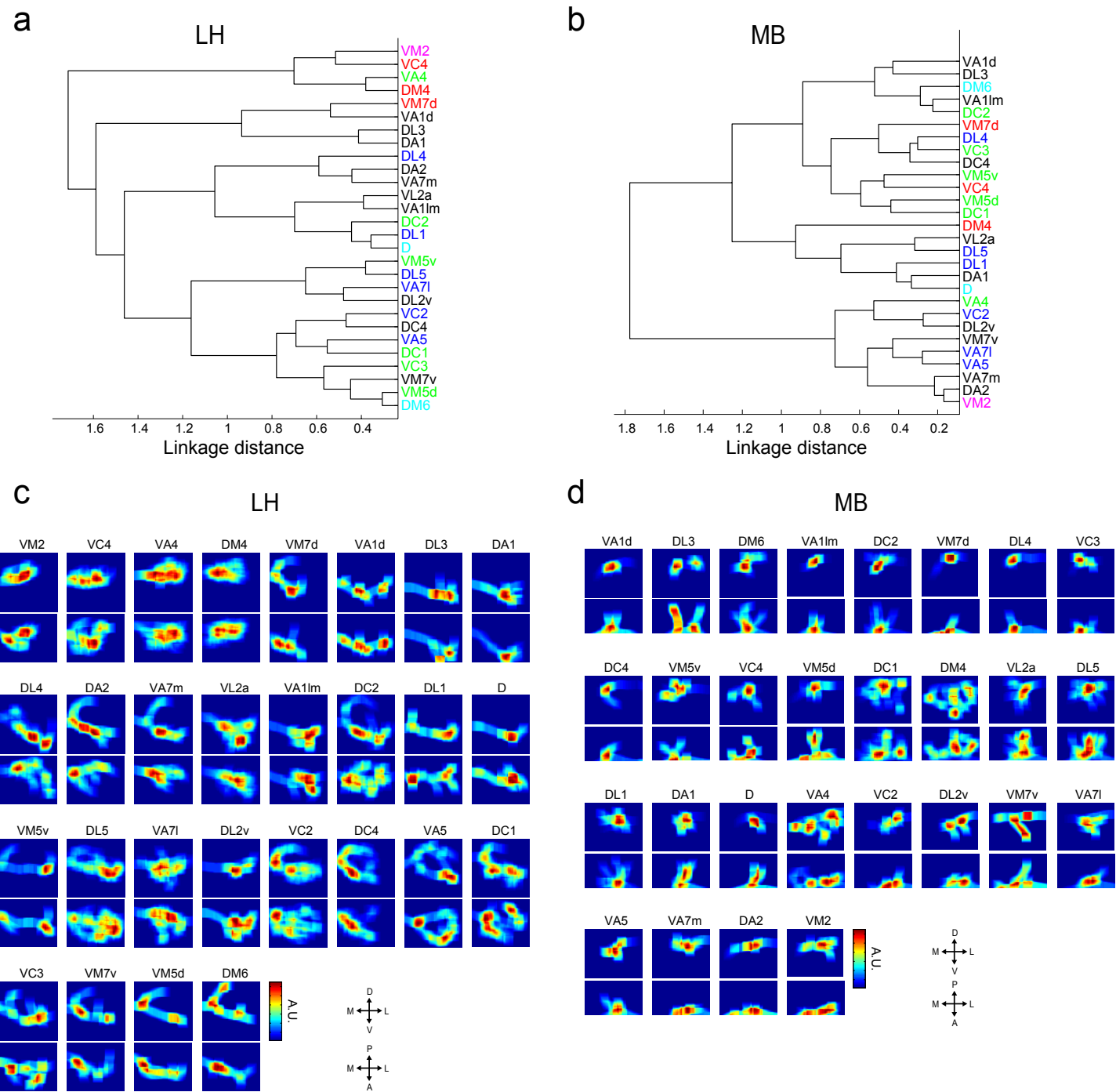

Supplement: Supplementary file 11 — Axonal density maps of PNs in the LH and in the MB. (a) Hierarchical cluster analysis for 28 PN classes based on the correlation distances between axonal density maps in the LH. (b) Hierarchical cluster analysis for 28 PN classes based on the correlation distances between axonal density maps in the MB. (c) The density maps of axonal projections of each PN class are visualized two-dimensionally in the LH. The pseudo color for each PN is scaled respectively. (d) The density maps of axonal projections of each PN class are visualized two-dimensionally in the MB. The pseudo color for each PN is scaled respectively. (PDF 755 kb) [file 12915_2017_389_MOESM11_ESM.pdf]

# Supplementary Figure 9

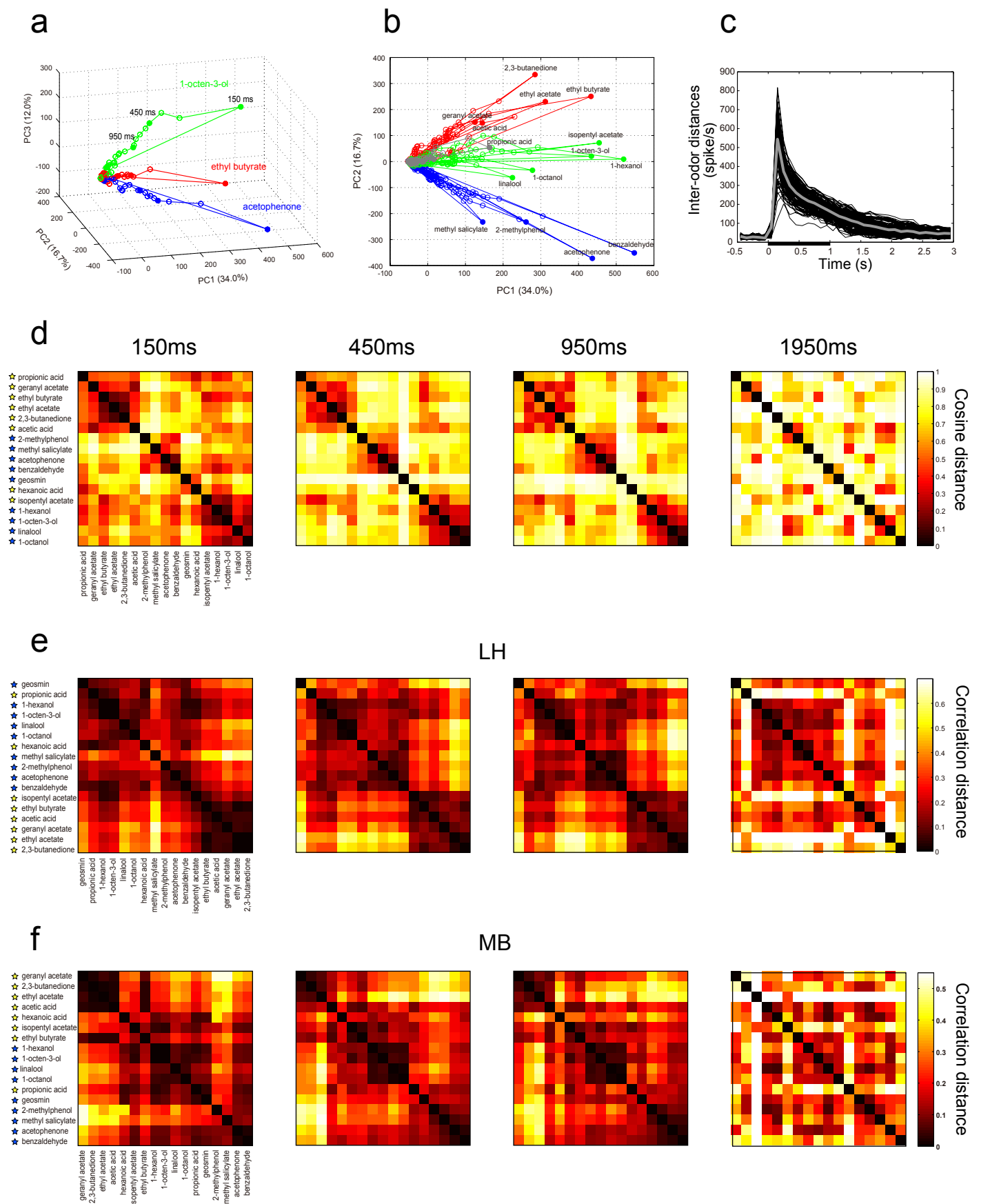

Supplement: Supplementary file 12 — Temporal dynamics of odor representation. (a) Represented trajectories of PN ensemble activities for three odors (red: ethyl butyrate, blue: acetophenone, green: 1-octen-3-ol) visualized in the three-dimensional PC space. Color is matched with Fig. 4a. Each trajectory is reconstructed with 100-ms steps indicated with circles, and filled circles indicate 150, 450, and 950-ms time frames. (b) Two-dimensional view of the odor response trajectories for all odors. The odors within the same cluster (colored in red, blue, green, and gray) in Fig. 4a have similar trajectories compared to those between different clusters. (c) Inter-odor distances between pairs of odors measured by ensemble PN odor responses with Euclidean distances. (d) Distance matrices of odor representations by PNs at different time frames. Odors are ordered in the same order as in Fig. 4b, to facilitate comparison to the pattern reconstructed with the mean firing rate for 1-s odor stimuli. Clustering of odors remain largely distinct during 1-s odor stimulus but disperse at 2 s. (e), (f) Distance matrices of odor representations in the LH (e) and MB (f) at different time frames. Odors are ordered in the same order as in Fig. 6b (for LH) and Fig. 7b (for MB) to facilitate comparison to the pattern reconstructed with the mean firing rate for 1-s odor stimuli. (PDF 994 kb) [file 12915_2017_389_MOESM12_ESM.pdf]
